# A siamese network with adaptive gated feature fusion for individual knee OA features grades prediction

**DOI:** 10.1038/s41598-021-96240-8

**Published:** 2021-08-19

**Authors:** Kang Wang, Xin Niu, Yong Dou, Dongxing Xie, Tuo Yang

**Affiliations:** 1 National Laboratory for Parallel and Distributed Processing, School of Computer, National University of Defense Technology, Changsha, 410073 China; 2 Department of Orthopaedics, Xiangya Hospital, Central South University, Changsha, 410008 China; 3 Department of Health Management Center, Xiangya Hospital, Central South University, Changsha, 410008 China

**Keywords:** Musculoskeletal abnormalities, Osteoarthritis, Radiography, Bone imaging

## Abstract

Grading individual knee osteoarthritis (OA) features is a fine-grained knee OA severity assessment. Existing methods ignore following problems: (1) more accurately located knee joints benefit subsequent grades prediction; (2) they do not consider knee joints’ symmetry and semantic information, which help to improve grades prediction performance. To this end, we propose a SE-ResNext50-32x4d-based Siamese network with adaptive gated feature fusion method to simultaneously assess eight tasks. In our method, two cascaded small convolution neural networks are designed to locate more accurate knee joints. Detected knee joints are further cropped and split into left and right patches via their symmetry, which are fed into SE-ResNext50-32x4d-based Siamese network with shared weights, extracting more detailed knee features. The adaptive gated feature fusion method is used to capture richer semantic information for better feature representation here. Meanwhile, knee OA/non-knee OA classification task is added, helping extract richer features. We specially introduce a new evaluation metric (top±1 accuracy) aiming to measure model performance with ambiguous data labels. Our model is evaluated on two public datasets: OAI and MOST datasets, achieving the state-of-the-art results comparing to competing approaches. It has the potential to be a tool to assist clinical decision making.

## Introduction

**Figure 1:**
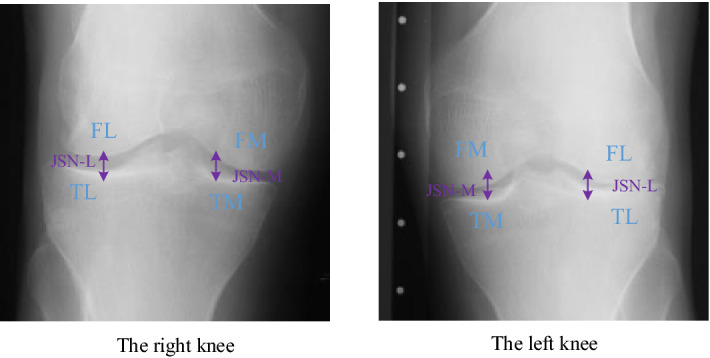
The specific features in knee images.

Knee osteoarthritis (OA)^1^ is a degenerative joint disease, mainly presenting osteophytes formation and knee joint space narrowing^2–4^. Severe cases may cause excruciating pain and even total joint replacement^5^. And the huge expense of knee treatment is surprising, even reaching 19000 euros one year for each patient^6^. Thus, early diagnosis and treatment are necessary for the defense of knee OA. Recently, the growth of the computer applications has achieved great success in medical engineering, so is for knee OA diagnosis. Computer-aided diagnosis^3^ reduces the subjectivity of assessing knee OA and achieves automatic knee OA diagnosis rapidly. Currently, the common computer-aided diagnosis is based on radiography (X-rays)^7^, which is the cheap and widely used medical imaging compared with other imaging^8,9^ (e.g., magnetic resonance imaging (MRI), ultrasound imaging, etc). The gold standard of predicting knee OA severity in X-rays is Kellgren-Lawrence (KL) grading system^10^, which includes KL0 (no OA), KL1 (Doubtful OA), KL2 (Minimal OA), KL3 (Moderate OA) and KL4 (Severe OA). However, KL grade is a composite score, which does not separately focus on individual features and lateral OA side/ medial OA side. Later, Osteoarthritis Research Society International (OARSI) atlas^11^ describes a feature-specific approach to grade knee OA severity. Specific features (see Fig. 1) include the lateral joint space narrowing (JSN-L), the medial joint space narrowing (JSN-M), the femoral lateral osteophytes (FL), the femoral medial osteophytes (FM), the tibial lateral osteophytes (TL) and the tibial medial osteophytes (TM), grades of which all contain four grades from Grade 0 to Grade 3. This provides a fine-grained knee OA severity assessment, palying an important role in supporting clinical decisions.

Up to now, several studies have demonstrated success in diagnosing knee OA KL-grade from X-rays, but merely a few studies about assessing individual knee OA features from X-rays exist. They usually locate knee joints firstly and then make subsequent diagnosis. Tiulpin et al.^12^ presented HOG and SVM method^13^ to detect knee joints and a 7-layer Siamese convolutional neural network to classify knee OA KL grades, having good performance in KL-grade prediction. However, the knee joint detection method via HOG and SVM is a traditional machine learning method, which is inferior to deep learning methods in the feature extraction. Although its KL-grade classification method considers more detailed local features by feeding image patches, it does not adopt more effective networks and feature fusion strategy for better feature representation. Moreover, Tiulpin et al. merely studied knee OA KL-grade prediction, ignoring individual knee OA features grades. In a later work^14^, Tiulpin et al. leveraged an ensemble model of SE-ResNet50 and SE-ResNext50 to simultaneously predict KL and OARSI grades in knee radiographs. It is computationally heavy due to ensembling. Random forest regression voting approach from the BoneFinder tool^14^ is applied to localize knee joints, which is also a traditional machine learning method with lower detection performance. Besides, it uses whole knee joint areas as input, neglecting the symmetry, richer semantic information and contrast features of knee joint parts. The individual knee OA features grades prediction performance should be further enhanced. In addition, these methods all use top1 accuracy to evaluate prediction performance, ignoring semi-quantitative labels and their ambiguity.

To solve problems above, we propose a SE-ResNext50-32x4d-based Siamese network with adaptive gated feature fusion strategy for individual knee OA features grades prediction.A deep learning method with two cascaded small multi-task networks is presented to localize initial knee joints, being able to extract more detailed knee features to enhance detection performance compared to traditional machine learning methods. Each obtained knee joint region is split into the left and right patches equally via its symmetry and fed into a deeper SE-ResNext50-32x4d-based Siamese network with shared weights, extracting more specific and richer local features than Tiulpin et al.’ methods^12,14^. Adaptive gate mechanism is embedded to fuse two parts’ features, which is helpful to capture valuable semantic information and more distinguishable contrast features of two parts compared with methods of Tiulpin et al.^12,14^. Furthermore, we put forward simultaneous assessment of eight tasks to learn more available features for prediction accuracy improvement, where the knee OA ($KL\ge 2$)/non-knee OA ($KL\le 1$) classification task is added compared with methods of Tiulpin et al.^12,14^. We also extend our model by integrating SE-ResNext50-32x4d-based Siamese network with two patches as input and SE-ResNext50-32x4d network with the whole knee joint region as input, which fuses local and global information of knee joint regions and further improves prediction performance. Besides, one new evaluation metric (i.e., the top$\pm 1$ accuracy) is introduced because of labels with ambiguity. Specifically, if it is a KL1 knee image and predicted as KL1, KL0 or KL2, the prediction is accepted as accurate. The top±1 accuracies of OARSI grades are the same. The main contributions in this paper are shown as follows:

1. To extract more effective knee features and enhance detection accuracy, a novel deep learning method with two cascaded small multi-task networks is proposed to localize knee joints.
2. A deeper SE-ResNext50-32x4d-based Siamese network with shared weights is first used to extract richer local features from two knee joints’ patches for grading individual knee OA features, making full use of knee joints’ symmetry.
3. An adaptive gated feature fusion method is designed to help capture more useful semantic information and better contrast features of two patches.
4. It is the first time to simultaneously evaluate eight tasks, where the knee OA ($KL\ge 2$)/non-knee OA ($KL\le 1$) classification task is added for promoting feature extraction.
5. We come up with a new evaluation metric (i.e., the top±1 accuracy) for assessing KL and OARSI grades prediction performance. And our proposed method achieves the state-of-the-art performance in grading individual knee OA features.

## Related works

Several classical studies include knee OA KL-grade diagnosis from X-rays^12,15–19^, individual knee OA features grades assessment from X-rays^20–22^, knee OA progression prediction^23^ and Magnetic Resonance Imaging (MRI) data analysis^8,9^. As for knee OA KL-grade diagnosis from X-rays, Shamir et al. introduced the WND-CHARM method^15–17^, which uses computer-aided analysis to diagnose early knee OA. Recently, deep learning methods have achieved great success in computer vision fields (e.g., automatic detection^24^, automatic segmentation^25^, image recognition^26^, video classification^27^, image retrieval^28^, etc.), directly extracting features from data and representing data more effectively compared with traditional approaches. Unsurprisingly, deep learning approaches also revolutionize the field of medical image analysis^29–33^. Antony et al.^18^ used Sobel horizontal image gradient features and SVM to localize knee joint regions. Pre-trained convolutional neural networks, such as VGG16^34^, VGG-M-128^35^ and CaffeNet^36^, via the ImageNet dataset^37^ are migrated to perform the fine-tuning on the knee OA KL-grade classification task. However, their knee joints localization method suffers a low detection accuracy. Thus, detected knee joint regions cannot be directly used for the subsequent diagnosis, and manually extracted knee joint regions are utilized. FCN-based method^38^ for knee joint localization was introduced by Antony et al.^19^, and a six-layer convolutional neural network with mean square error loss and the cross-entropy loss is cascaded to predict knee OA KL grades. However, it is time-consuming for FCN-based method to generate binary images by segmenting knee regions from each pixel. And the prediction performance of knee OA severity should be further improved. Later, Tiulpin et al.^12^ utilized HOG and SVM method^13^ to detect knee joints. Knee joints are divided into symmetric image blocks, which are sent into a 7-layer Siamese convolutional neural network to diagnose knee OA KL grades. The knee joint localization accuracy should be further promoted due it is also a traditional machine learning approach. For KL grades classification, although it considers the symmetry of knee joint and extracts more detailed local features, it does not use deeper network structure and more effective feature fusion strategy to extract better contrast and semantic information for higher accuracy. Chen et al.^39^ used YOLOv2^40^ architecture to detect knee joints and proposed a novel ordinal loss, which replaces cross-entropy loss and is combined with VGG19^34^ model to achieve satisfactory knee OA severity grading prediction performance. Mikhaylichenko et al.^41^ applied Single Shot Detector (SSD)^42^ model for knee joint localization and utilized DenseNets^43^ to assess grades of knee OA. These two works use the whole knee region as input, ignoring local features and semantic information of left and right parts of each knee area. Moreover, above studies merely predict knee OA KL grades, ignoring individual knee OA features. So far, merely a few works have studied individual knee OA features assessment from X-rays. Osteoarthritis Research Society International (OARSI) atlas^44^ is the feature-specific approach to grade knee OA severity by grading features (i.e., JSN-L, JSN-M, FL, FM, TL, TM.). The automatic analysis of individual knee OA features was firstly reported by Oka et al.^20^. Later, Thomson et al.^21^ utilized shape and texture descriptors to evaluate the presence of osteophytes and knee OA ($KL\ge 2$). However, the test set they used is relatively small compared to other OA studies. Antony et al.^22^ proposed a CNN-based approach for simultaneous analysis of KL and OARSI grades, the prediction accuracy of which needs to be further improved. Tiulpin et al.^14^ presented an ensemble model of SE-ResNet50 and SE-ResNext50 to simultaneously assess KL and OARSI grades in knee radiographs, which is time-consuming because of ensembling. They put the whole knee joint into training models without considering knee joints’ symmetry and richer semantic information. They applies random forest regression voting algorithm^14^ for knee detection, which is a traditional machine learning method with lower detection performance. Thus, to improve knee joint detection accuracy, a deep learning method with two-level cascaded multi-task network is proposed. To extract more detailed local features and more meaningful semantic information, a deeper SE-ResNext50-32x4d-based Siamese network with shared weights and adaptive gated feature fusion method is proposed to process knee joint patches and simultaneously assess more tasks.

## Methods

This study was approved by the Institutional Reviewing Board (IRB) of National Laboratory for Parallel and Distributed Processing, School of Computer, National University of Defense Technology, and Xiangya Hospital, Central South University with informed consent obtained from all participants prior to the start of the study. All methods were carried out in accordance with relevant guidelines and regulations. Our experimental data are publicly available, which were approved by the institutional review board of the University of California San Francisco and obtained the informed consent of all subjects participating in the study. It is described in detail in datasets and data preprocessing part.

**Figure 2:**
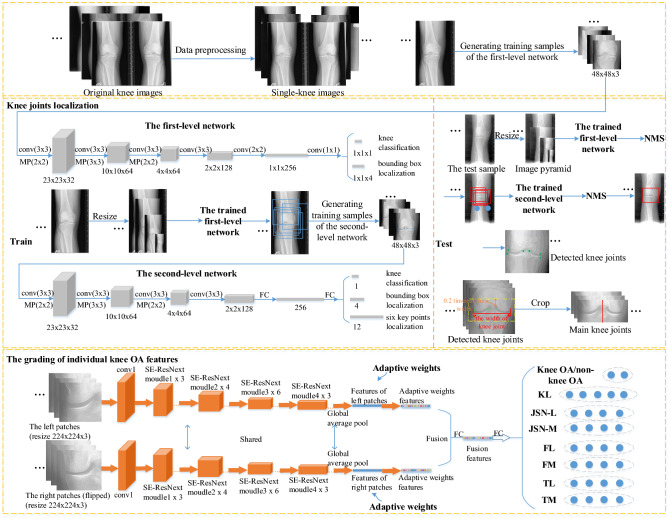
The whole process of the proposed method.

The whole process of our proposed method is shown as Fig. 2. Original double-knee images are processed and divided into single-knee images. A two-level cascaded multi-task network is trained and tested for localizing knee joints of single-knee images. To further reduce redundancy, located knee joints are cropped again to generate main knee joint regions, each of which is divided into left and right patches with its symmetry. After the right image patch is horizontally flipped, then two patches are fed into a Siamese SE-ResNext50-32x4d Network with shared weights. Adaptive gate mechanism strategy is exploited to fuse contrast features from two patches before fully connected (FC) layers. Fused features are put into subsequent FC layers for assessing individual knee OA features grades. Eight tasks are simultaneously assessed for the first time, where knee OA ($KL\ge 2$)/non-knee OA ($KL\le 1$) classification task is added to further enhance feature extraction.

### Datasets and data preprocessing

We utilized two publicly available knee X-ray datasets: the OAI dataset (https://nda.nih.gov/oai/) and the MOST dataset (http://most.ucsf.edu). The OAI dataset we obtained contains the X-ray data from 4796 subjects and their seven follow-up examinations (i.e., baseline, 12-month, 24-month, 36-month, 48-month, 72-month and 96-month). The MOST dataset we obtained includes the X-ray data from 3026 participants and their five follow-up examinations (i.e., baseline, 15-month, 30-month, 60-month and 84-month), which does not belong to the OAI dataset. Both datasets include double-knee X-rays from men and women aged between 50-79 and 45-79 years old. Because data with missing labels exist in two databases, we select data with KL labels and OARSI scorings for our experiments.

Each double-knee X-ray with DICOM format contains the left and right knee images in two datasets. Due to the influence of different illumination during shooting, some X-rays have the bright background and dark knees, and some have the opposite. Data preprocessing is first performed to unify knee X-rays, the specific process of which is shown in supplementary Fig. 1. First of all, original X-ray knee images with bright background and dark knees are chosen to perform pixel inversion, which means that these X-rays are transformed into images with dark background and bright knees. Therefore, all double-knee images are turned to dark background and bright knees. Then we divide each double-knee image into two single-knee images. Meanwhile, each DICOM format image is converted into the 8-bit uint image. Finally, the histogram equalization is used on all single-knee images. In the end, 24319 and 18634 single-knee images are generated on the OAI dataset and the MOST dataset, respectively.

### Knee joints localization

A two-level cascaded multi-task network is built to localize knee joint regions, inspired by MTCNN method^45^. As shown in Fig. 2, the knee joints localization network contains two small neural networks. In the first-level network, three convolutions and maximum pooling operations are sequentially performed first. Then three convolution operations follow. In the end, one-dimensional vector about knee/non-knee classification and 4-dimensional bounding box regression vectors of candidate knee joint regions are output. The second-level network also performs three convolutions and maximum pooling operations on input images. Then one convolution operation and two FC layers are connected behind. Finally, one-dimensional vector about knee/non-knee classification, 4-dimensional bounding box regression vectors of detected knee joints and 12-dimensional vectors of six key points are output.

The whole training target of our localization model is as (1), where *N* is the number of training samples. $\alpha _{\text {det}}$, $\alpha _{\text {box}}$ and $\alpha _{\text {key points}}$ stand for the importance of knee/non-knee classification task, bounding box regression task and key points localization task, respectively. $\text {Loss}_i^{\text {det}}$, $\text {Loss}_i^{\text {box}}$ and $\text {Loss}_i^{\text {key points}}$ represent the cross-entropy loss of knee/non-knee classification task, the Euclidean loss of bounding box regression task and the Euclidean loss of key point localization task for the *i*-th sample, respectively. In the first-level network, we set $\alpha _{\text {det}}=1$, $\alpha _{\text {box}}=0.5$ and $\alpha _{\text {key points}}=0$. In the second-level network, we set $\alpha _{\text {det}}=0.8$, $\alpha _{\text {box}}=0.6$ and $\alpha _{\text {key points}}=1.5$. The training process of our localization model is concretely introduced in supplementary information.

1 $$\begin{aligned} \min {\sum _{i=1}^N}\{\alpha _{det}{\text {Loss}_i^{\text {det}}}+{\alpha _{box}}{\text {Loss}_i^{\text {box}}}+{\alpha _{key points}}{\text {Loss}_i^{\text {key points}}}\}. \end{aligned}$$

During the test stage, each single-knee image is resized with different scales to generate the image pyramid. The image pyramid is put into the trained first-level network, generating some candidate knee joint regions. Then highly overlapped candidate regions are merged by the non-maximum suppression (NMS). All reserved candidate regions are fed to the trained second-level network. The second-level network further rejects a few false candidates. Then, NMS is also carried out. Finally, the knee joints and six key points of single-knee images are detected.

### The grading of individual knee OA features

In this subsection, we will specifically describe our SE-ResNext50-32x4d-based Siamese network via adaptive gated feature fusion for grading individual knee OA features in X-rays. Firstly, we perform data processing to further crop detected knee joint areas via six key points and flexibly obtain main knee joint areas for further reducing redundancy. Then, we divide each main knee joint region into left and right patches according to its symmetry and flip the right patch horizontally, which are fed into a SE-ResNext50-32x4d-based Siamese network with shared weights. Finally, adaptive gated feature fusion is used to fuse more distinguishable contrast features of two patches before FC layers.

#### Data processing

Tiulpin et al.^12^ selected a fixed position and size area from each located knee joint region for subsequent diagnosis. However, the knee joint width of each person is different. If knee joint areas are cropped according to the fixed number of pixels, which will lead to inaccurate repositioning of knee joint regions. Relocating initial knee joint areas via the ratio of the knee joint width for each person is able to increase the flexibility and accuracy of knee joints relocation. Here, we regard the difference between the maximum and minimum abscissas of the six key points as the knee joint width. In Fig. 2, for each detected knee joint from knee joints localization model, the maximum and minimum ordinates of the six key points are first found. Then the maximum ordinate increases by 0.2 times of knee joint width as the top of the main knee joint. The minimum ordinate is reduced by 0.2 times of knee joint width as the bottom of the main knee joint. Finally, main knee joints are obtained with same width and cropped height compared to initially detected knee joint regions.

#### The SE-ResNext50-32x4d-based Siamese network

The effective SE-ResNext50-32x4d-based Siamese network we proposed consists of two SE-ResNext50-32x4d branches. The SE-ResNext50-32x4d branch is built up by a stack of modules, as shown in Fig. 2, where a basic SE-ResNext module (see in supplementary Fig. 4 (d)) includes the ResNext module and the Squeeze and Excitation (SE)^46^ module.

The ResNext block proposed by Xie et al.^47^ is a residual block^48^ with split-transform-merge strategy in Inception^49,50^. The ResNext block performs a set of transformations, as shown in supplementary Fig. 4 (a), where each transformation is set as the bottleneck shaped architecture^47^. Firstly, the vector *x* is broken up into low-dimensional embeddings before the first $1\times 1$ layers. Then transformations are performed for low-dimensional embeddings. Finally, all transformations are aggregated. The output of ResNext block can be represented as (2), where $f_{i}()$ is a function that divides *x* into a low-dimensional embedding and transforms it. *C* is defined as cardinality^51^, which is the number of aggregated transformations. Here, C is set as 32 and the width of the bottleneck is 4 in SE-ResNext50-32x4d model. As supplementary Fig. 4 (b) shown, when the ResNext module uses grouped convolutions^52^, it becomes more simple and equivalent to Fig. 4 (a) in supplementary information. In the module, 32 $1\times 1$ layers are replaced by a $1\times 1$, 128-d layer. Then 32 groups of convolutions are performed in the grouped convolutional layer and finally concatenated as the output.

2 $$\begin{aligned} y=x+\sum _{i=1}^{C}f_{i}(x). \end{aligned}$$

The Squeeze and Excitation (SE) unit is introduced by Hu et al.^46^ and used to improve the representational capacity of the network by explicitly establishing channel interdependencies of features maps. The SE block is able to selectively highlight value features and suppress less useful ones by feature recalibration, the structure of which is illustrated in supplementary Fig. 4 (c). Features P are obtained after one or a series of convolution operations. Then a corresponding SE block follows. Firstly, features P are passed through a squeeze operation, which uses the global average pooling. The feature maps across spatial dimensions are aggregated and channel descriptors are generated as (3), where $c = 1,2,\ldots C$, $z_{c}$ represents the *c*-th channel descriptor. Then an excitation operation follows, which aims to acquire dependencies on channels in (4). The first FC layer that is a dimensionality-reduction layer with parameters $W_{1}$ with reduction ratio *r* is performed, $W_{1} \in R_{C_{r}\times C}$. A ReLU^53^ function follows and is represented as $\tau$. The second FC layer is a dimensionality-increasing layer with parameters $W_{2}$ and $W_{2} \in R_{C\times C_{r}}$. A sigmoid function as a simple self-gating mechanism is used to produce a corresponding weight for each channel. In the end, the output of the SE block is generated by weighting features P as (5), where ${\widetilde{P}}_{c}$ represents the weighted feature of the *c*-th channel.

3 $$\begin{aligned}&z_{c}=F_{sq}(p_{c})=\frac{1}{H \times W}\sum _{i=1}^{H}\sum _{j=1}^{W}p_{c}(i,j). \end{aligned}$$

4 $$\begin{aligned}&w=F_{ex}(z,W)=\sigma (W_{2}\tau (W_{1}z)). \end{aligned}$$

5 $$\begin{aligned}&{\widetilde{P}}_{c}=F_{scale}(p_{c},w_{c})=w_{c}\cdot p_{c}. \end{aligned}$$

#### The adaptive gated feature fusion method

Inspired by Zhang et al.^54^ who proposed a gated multimodal fusion method, we propose an adaptive gated feature fusion method. First of all, we extract features of the left and right knee joint image patches through a SE-ResNext50-32x4d-based Siamese network for selecting useful features and suppressing useless features. We adopt a gate mechanism to adaptively decide weights of the left and the right knee patch features for extracting more meaningful contrast features. Then the fused features are obtained as (6).

6 $$\begin{aligned} g=\sigma \left( W_{g}\left( f_{left}\bigoplus f_{right}\right) \right) , f = gf_{left}+(1-g)f_{right}. \end{aligned}$$

where $$W_{g}$$ is the network parameter. $$f_{left}$$ and $$ f_{right}$$ stand for the features of left and right knee patches extracted from the SE-ResNext50-32x4d-based Siamese network, respectively. $$\bigoplus $$ is the concatenating operation, $$\sigma $$ is the sigmoid function, *g* is the weight applied to the $$f_{left}$$ feature, *(1-g)* is the the weight applied to the $$f_{right}$$ feature. *f* is the fused feature of the left and the right knee patches. This is an adaptive learning method, which learns weighted features of each image patch and the correlation between two symmetrical patches. The key regions that tasks pay more attention to are learned by the proposed model, which can improve the accuracy of assessing individual knee OA features grades. Eight tasks are predicted simultaneously, including predictions of KL grades, JSN-L grades, JSN-M grades, FL grades, FM grades, TL grades and TM grades, and knee/non-knee OA classification that we first proposed to add.

### Implementation details

#### Knee joint detection model

Training parameters of the first-level network are set as follows: the *epoch* is 10, the *learning rate (lr)* is 0.001, and the *batch_size* is 500. The training parameters of the second-level network are set as follows: the *epoch* is 10, the *lr* is 0.0001, and the *batch_size* is 500.

#### Grading individual knee OA features model

We set the training parameters on both datasets as follows^14^: For the first two training epochs, only the FC layers are trained with *lr* of 0.01. Subsequently, the whole network is trained with *lr* of 0.001. *lr* is switched to 0.0001 from the fourth epoch. 20 epochs and Adam optimizer^55^ are used in all experiments. To avoid over-fitting problems^56^, we use data augmentations^34,48^: illumination contrast enhancement, gamma correction, rotation and translation, etc. Besides, we also use weight decay of 0.0001 and dropout of 0.5 that is inserted before each FC layer.

Our experiments are deployed on the Ubuntu 16.04 platform, depending on Python 3.6, PyTorch 0.4 and 2080Ti GPU.

## Results

### Experimental results and analyses of knee joint detection

#### Experimental results

**Figure 3:**
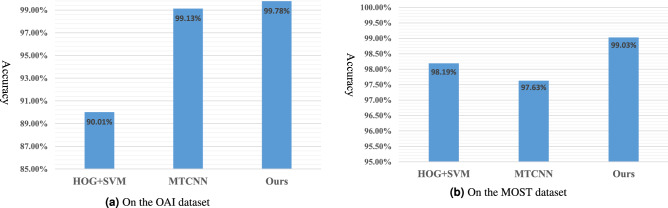
The detection accuracy comparison of knee joints with different methods on the OAI and MOST datasets.

The knee joints localization model achieves 99.85% accuracy in validation set, i.e., 1360 out of 1362 are detected. Test results of two datasets are shown in Fig. 3. Figure 3a shows that the proposed knee joints localization method is able to detect more knee joints than the HOG+SVM^13^ and MTCNN^45^ methods on the OAI dataset. The average detection accuracy is 99.78%, which is 0.65% higher than the MTCNN^45^ method, and 9.77% better than the HOG+SVM^13^ method. From the Fig. 3 (b), the average detection accuracy of our method is 99.03% on the MOST dataset, which is 0.84% better than that (98.19%) of the HOG+SVM^13^ method and 1.4% higher than the MTCNN^45^ method. Therefore, our knee joints localization algorithm shows superior performance. Finally, 24265 and 18454 knee joint regions from the OAI dataset and the MOST dataset are detected, respectively, which can be directly used for the subsequent assessment of individual knee OA features grades.

#### Analyses

Compared with the traditional approach (e.g., HOG+SVM), our proposed model is a deep learning method, having the ability to extract more discriminative and detailed features. Meanwhile, our two-level cascaded framework is superior to original MTCNN model that consists of three cascaded networks. In our model, extra convolution layers are added in the first-level network to extract richer knee joint features, improving the detection performance of the first-level network. In the end, our designed two-level cascaded network can defeat three-level one (i.e., MTCNN).

### Experimental results and analyses of grading individual knee OA features

Detected knee images are randomly divided into training, validation, and test sets with a ratio of 5:1:3, whose KL distribution keeps consistent. Specific description of experimental data is presented in supplementary Table 1 and Table 2. Finally, 24265 detected knees from the OAI dataset are divided into 13472 training sets, 2732 validation sets and 8061 test sets. 18454 detected knees from the MOST dataset are divided into 10244 training sets, 2048 validation sets and 6162 test sets. These detected knee joint regions are further relocated to generate main knee joint areas.

**Table 1:** Performance comparison between the proposed method and other methods on the OAI dataset.

Methods	Top1							
	knee OA/non-knee OA	KL	FL	FM	TL	TM	JSN-L	JSN-M
Antony et al., 2017^19^	–	46.62%	–	–	–	–	–	–
Tiulpin et al., 2018^12^	–	63.79%	–	–	–	–	–	–
Chen et al., 2019^39^	–	71.69%	–	–	–	–	–	–
Mikhaylichenko et al., 2021^41^	–	73.02%	–	–	–	–	–	–
SE-ResNet-50^14^	–	72.96%	70.56%	70.54%	74.30%	68.12%	91.20%	78.55%
SE-ResNext50-32x4d^14^	–	75.74%	72.75%	72.91%	76.79%	71.69%	91.48%	79.92%
Ensemble^14^	–	76.99%	73.24%	73.04%	76.89%	71.93%	**92.02**%	**81.26**%
Ours	88.48%	76.32%	74.80%	74.73%	76.81%	72.73%	91.11%	80.00%
Ours (Ens.)	**89.60**%	**78.18**%	**75.05**%	**74.82**%	**77.74**%	**73.60**%	91.39%	80.36%

Methods	Top±1							
	–	KL	FL	FM	TL	TM	JSN-L	JSN-M
Antony et al., 2017^19^	–	84.74%	–	–	–	–	–	–
Tiulpin et al., 2018^12^	–	88.54%	–	–	–	–	–	–
Chen et al., 2019^39^	–	**97.30**%	–	–	–	–	–	–
Mikhaylichenko et al., 2021^41^	–	95.57%	–	–	–	–	–	–
SE-ResNet-50^14^	–	95.89%	93.44%	92.74%	95.89%	97.26%	97.84%	97.98%
SE-ResNext50-32x4d^14^	–	96.22%	94.52%	94.02%	96.07%	97.43%	98.02%	97.90%
Ensemble^14^	–	96.35%	94.42%	94.05%	96.17%	97.72%	**98.05**%	**98.08**%
Ours	–	96.63%	**95.31**%	94.59%	96.91%	97.67%	97.89%	97.75%
Ours (Ens.)	–	97.01%	95.12%	**94.68**%	**97.05**%	**97.77**%	97.85%	97.85%

**Table 2:** Performance comparison between the proposed method and other methods on the MOST dataset.

Methods	Top1							
	knee OA/non-knee OA	KL	FL	FM	TL	TM	JSN-L	JSN-M
Antony et al., 2017^19^	–	49.48%	–	–	–	–	–	–
Tiulpin et al., 2018^12^	–	68.73%	–	–	–	–	–	–
Chen et al., 2019^39^	–	75.56%	–	–	–	–	–	–
Mikhaylichenko et al., 2021^41^	–	73.43%	–	–	–	–	–	–
SE-ResNet-50^14^	–	74.39%	79.32%	78.33%	76.87%	71.19%	93.20%	84.00%
SE-ResNext50-32x4d^14^	–	75.22%	78.98%	78.72%	78.04%	73.11%	93.43%	84.16%
Ensemble^14^	–	76.13%	80.31%	79.23%	78.42%	73.56%	**93.69**%	**84.83**%
Ours	92.58%	76.94%	**82.05**%	**80.54**%	**80.12**%	76.32%	93.54%	83.77%
Ours (Ens.)	**92.84**%	**77.86**%	80.88%	79.91%	79.52%	**76.71**%	93.31%	84.19%

Methods	Top±1							
	–	KL	FL	FM	TL	TM	JSN-L	JSN-M
Antony et al., 2017^19^	–	73.06%	–	–	–	–	–	–
Tiulpin et al., 2018^12^	–	90.28%	–	–	–	–	–	–
Chen et al., 2019^39^	–	98.09%	–	–	–	–	–	–
Mikhaylichenko et al., 2021^41^	–	95.67%	–	–	–	–	–	–
SE-ResNet-50^14^	–	98.26%	93.72%	92.16%	94.55%	97.73%	98.17%	98.18%
SE-ResNext50-32x4d^14^	–	98.20%	95.16%	93.28%	96.45%	97.84%	98.26%	98.26%
Ensemble^14^	–	98.43%	94.64%	93.20%	95.80%	97.97%	98.30%	98.28%
Ours	–	**98.59**%	**95.55**%	**94.06**%	**96.87**%	**98.15**%	**98.33**%	98.21%
Ours (Ens.)	–	98.12%	94.86%	93.72%	96.59%	98.10%	98.20%	**98.31**%

#### Comparison with state-of-the-arts

Table 1 and Table 2 show experimental results of grading individual knee OA features, where ours (Ens.) represents an ensemble model (see supplementary Fig. 5) of SE-ResNext50-32x4d and SE-ResNext50-32x4d-based Siamese network for eight tasks. As for the single model on the OAI dataset, Table 1 shows that our proposed method (ours) is superior to methods proposed by Antony et al.^19^, Tiulpin et al.^12^, Chen et al.^39^, Mikhaylichenko et al.^41^, SE-ResNet-50^14^ model and SE-ResNext50-32x4d^14^ model in most cases for top1 accuracy; As for the ensemble model on the OAI dataset, ours (Ens.) is better than ensemble model^14^ except for JSN-L and JSN-M in top1 and top±1 accuracy. Table 1 clarifies that the optimal top1 accuracy of assessing knee OA/non-knee OA, KL grades, FL grades, FM grades, TL grades, TM grades, JSN-L grades and JSN-M grades are 89.60%, 78.18%, 75.05%, 74.82%, 77.74%, 73.60%, 92.02% and 81.26%, respectively; 97.30%, 95.31%, 94.68%, 97.05%, 97.77%, 98.05% and 98.08% are best top±1 prediction accuracy of KL grades, FL grades, FM grades, TL grades, TM grades, JSN-L grades and JSN-M grades on the OAI dataset, most of which are obtained under ours (Ens.). From Table 2, we can observe that ours and ours (Ens.) both outperform previous state-of-the-art models in top1 and top±1 accuracy in most cases. The highest top1 accuracy of predicting knee OA/non-knee OA, KL grades, FL grades, FM grades, TL grades, TM grades, JSN-L grades and JSN-M grades are 92.84%, 77.86%, 82.05%, 80.54%, 80.12%, 76.71%, 93.69% and 84.83%, respectively, which are mainly generated by our proposed methods except for JSN-L grades and JSN-M grades. Our proposed single model reaches the best top±1 accuracy of 98.59%, 95.55%, 94.06%, 96.87%, 98.15%, 98.33% in grading KL, FL, FM, TL, TM, JSN-L, respectively. And the best top±1 accuracy of 98.31% in grading JSN-M is achieved under ours (Ens.) method. The single model we proposed even surpasses the ensemble model^14^ in most cases on two databases. We also evaluate our proposed algorithms from Kappa and MSE metrics as shown in supplementary Table 3 and Table 4, where our proposed methods have higher Kappa and lower MSE than existing state-of-the-arts in most cases. In order to further verify the effectiveness of our algorithm, we extend experiments, using the OAI dataset as the training set and the MOST dataset as the test set. It avoids the influence of knee images with different months of the same person appearing in the training set and the test set. Supplementary Table 5 and Table 6 demonstrate that our methods outperform advanced works, having higher top1 accuracy, top±1 accuracy, Kappa and lower MSE in most cases. Thus, we can conclude that our proposed methods have the state-of-the-art performance, which extract more useful, richer features and semantic information, improving prediction performance in grading individual knee OA features.

#### Ablation study

To verify the effectiveness of each module, ablation study is conducted. SE-ResNext50-32x4d model using the whole knee joint as input is our baseline, which grades seven tasks (i.e., KL and OARSI). Supplementary Table 7 and Table 8 illustrate their experimental results, where we can find that Siamese SE-ResNext50-32x4d (baseline+Siamese) model exceeds baseline in most cases in terms of top1 and top±1 accuracy on the OAI and MOST datasets. Thus, it proves that two image blocks as input are beneficial to extract more sufficient local features than the whole knee joint as input, enhancing the classification performance. When we add knee OA/non-knee OA classification task on the basis of Siamese SE-ResNext50-32x4d (baseline+Siamese) model, they also show that adding prediction task obtains higher top1 accuracy and top±1 accuracy than baseline+Siamese model on two datasets. Obviously, adding knee OA/non-knee OA binary classification in the final prediction layer is helpful to improve prediction performance of individual knee OA features grades by facilitating richer knee OA features extraction. In supplementary Table 7, the top1 accuracy of assessing individual knee OA features via our proposed method that applies adaptively gated feature fusion module is superior to that of ignoring this module under most circumstances on the OAI dataset. Meanwhile, supplementary Table 8 describes that our method has better performance in all top1 accuracy compared with baseline+Siamese+knee OA/non-knee OA task. The top±1 accuracy of our method is higher than that of baseline+Siamese+knee OA/non-knee OA task apart from for FM grades prediction. Therefore, adaptively gated feature fusion module further boosts performance of individual knee OA features assessment, which highlights more valuable contrast features and semantic information of key regions.

**Figure 4:**
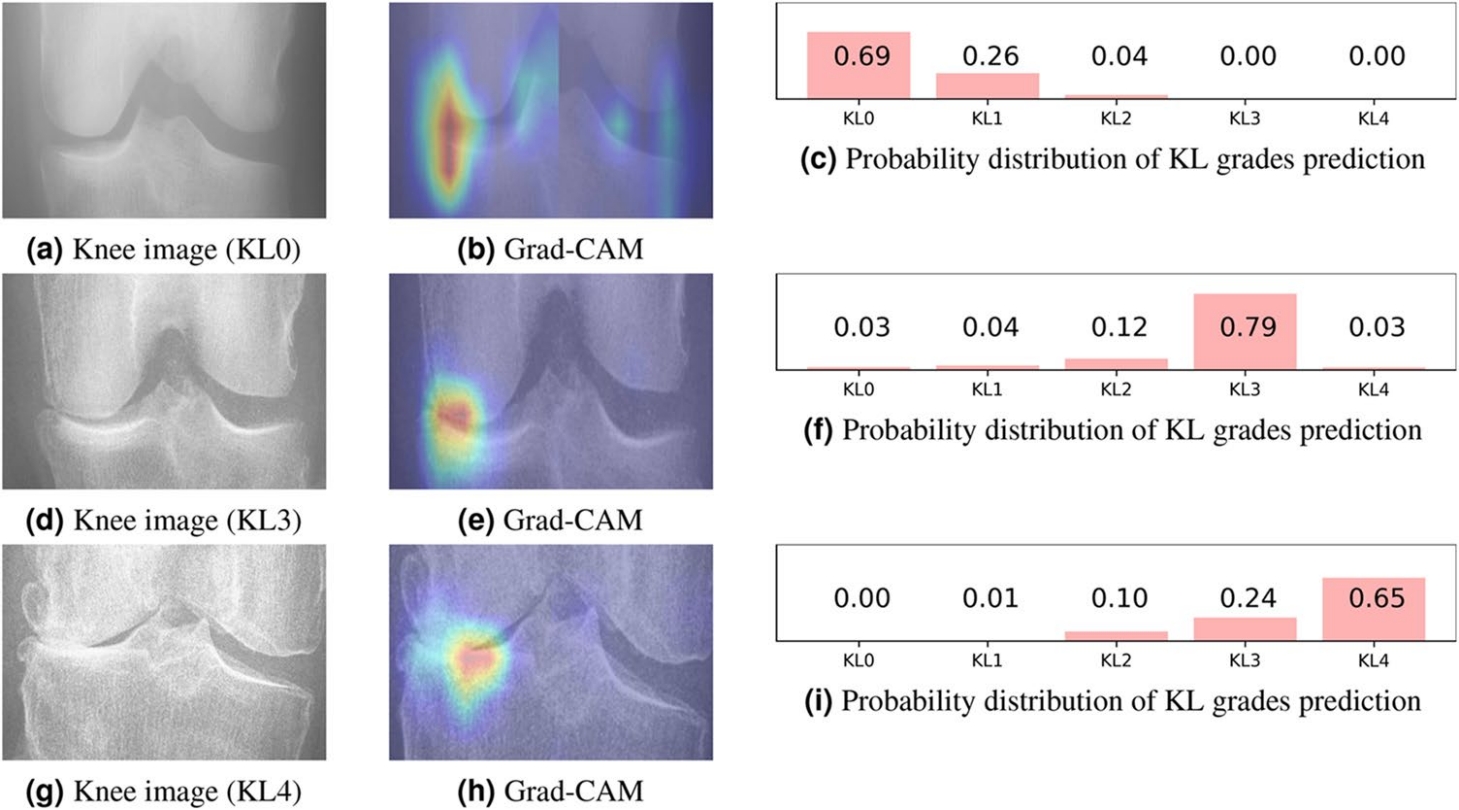
Examples of applying the Grad-CAM algorithm to our proposed model and probability distribution for KL grades prediction.

#### Analyses

Above ablation study demonstrates the effectiveness of each module in our proposed method. Compared with single-task methods (i.e., Antony et al.^19^, Tiulpin et al.^12^, Chen et al.^39^, Mikhaylichenko et al.^41^) that merely predict knee OA KL grades, our proposed methods not only realize fine-grained knee OA features grades prediction, but also achieve better classification performance in KL grades prediction, mainly presenting higher top1 accuracy, top±1 accuracy, Kappa coefficient and lower MSE in KL prediction. Additionally, we observe that Chen et al.’s method^39^ shows superior performance in top±1 accuracy due that its ordinal loss considers the closeness between different categories. Approaches (i.e., SE-ResNet-50, SE-ResNext50-32x4d and ensemble models) proposed by Tiulpin et al.^14^ are currently advanced in grading individual knee OA features. Our proposed methods have obvious superiority over methods of Tiulpin et al.^14^, acquiring higher top1 accuracy, top±1 accuracy, Kappa coefficient and lower MSE in most cases. We analyse the reasons as follows: (1) The SE-ResNext50-32x4d network is a deeper and more discriminative architecture, where SE-ResNext module helps to capture value features and suppress meaningless ones. (2) A Siamese network with SE-ResNext50-32x4d backbone can extract more sufficient local features since the left and right image patches are passed to SE-ResNext50-32x4d respectively. (3) Multi-task prediction can promote each other due to correlation among knee OA/non-knee OA, OARSI and KL, making the model pay more attention to osteophytes and joint spaces and extracting more critical features. (4) The adaptive gated feature fusion method is used for feature fusion of two parts, further filtering features of two parts to emphasize useful information and suppress the useless information by adaptive weighting. (5) We also propose an ensemble model consisting of two networks, where one network uses the whole knee joint region as input and the other one utilizes two parts of knee joint area as input, taking into account global and local information of knee joint regions. Moreover, confusion matrices for the KL, OARSI grades prediction and knee OA/non-knee OA binary classification prediction tasks are displayed from Fig. 6 to Fig. 20 in supplementary information, where predicted results for each grade of each task are shown. From supplementary Fig. 6 and Fig. 7, we can find that KL0, KL3 and KL4 are easier to be distinguished, however, there exists a higher confusion between KL1 and KL2 due to their unclear clinical symptoms. Similarly, it is more difficult to identify Grade 1 and Grade 2 than Grade 0 and Grade 3 for osteophytes grades prediction. Osteophytes of Grade 0 and Grade 3 have more distinct features. Supplementary Figure 16 to Figure 19 clarify that the classification performance is better at each grade prediction of joint space narrowing, and the overall performance is higher compared with osteophytes grades prediction. It seems that joint space narrowing grades are easier to diagnose than osteophytes grades because characteristics of joint space narrowing are more intuitive than osteophytes. Nevertheless, there still exists confusion among Grade 0, 1, and 2 due to the uncertain degree of knee joint space narrowing. Moreover, we provide additional information of our proposed model for helping physicians make clinical decisions, such as attention maps, the probability distribution of KL grades prediction, etc. (see in Fig. 4). It can be seen that our proposed model mainly focuses on abnormal osteophytes and knee joint spaces, and has high confidence in prediction. Thus, our method has the potential to be a tool to assist clinical decision making.

## Discussion

In this study, we propose a SE-ResNext50-32x4d-based Siamese network with adaptive gated feature fusion strategy to assess individual knee OA features grades. Based on results above, we conclude the following observation and discussions:

1. Two cascaded small multi-task networks are designed to locate knee joints, the average detection accuracy of which achieves 99.78% on the OAI dataset and 99.03% on the MOST dataset. Our detection model has capability of extracting more discriminative features compared with previous methods, enjoying obvious superiority. Thus, the proposed detection approach is an effective tool for locating knee joints, which are applicable for subsequent diagnosis.
2. As well known, the features of knee OA are concentrated around the knee joint spaces. The original located knee joints may contain some regions that contribute nothing. To reduce redundancy, we further crop located knee joints via six key points to generate main knee joints.
3. A SE-ResNext50-32x4d-based Siamese network is first to be used to grade individual knee OA features, where ResNext module and SE module help to capture more useful features from knee images. Furthermore, located whole knee joints are divided into two patches according to their symmetry, which are fed into our Siamese network with shared weights to extract more detailed features.
4. An adaptive gated strategy is applied to the feature fusion layer to further suppress useless information and highlight valuable information, which helps to capture richer semantic information and obtain better contrast features of two patches.
5. In order to fully extract knee joints features, we add the knee OA(KL$\ge$2)/non-OA(KL$\le$1) binary classification task to the other seven tasks we predicted simultaneously. The binary classification of knee OA/non-OA not only enhances other tasks’ prediction performance but also is vital for doctors’ preliminary clinical diagnosis.
6. We introduce a new evaluation metric that is top±1 accuracy to assess KL and OARSI grades. Due that knee OA progression is successive and expert evaluation is subjective, discrete labels of KL and OARSI have ambiguity. Therefore, we propose one new standard that the prediction belonging to true label’s adjacent grades is also regarded as an accurate one. To verify our methods, we compare our proposed methods of grading individual knee OA features grades with existing methods, our proposed method achieves promising results under different evaluation metrics.

Here, we merely consider the KL, OARSI grades prediction (i.e., JSN-L, JSN-M, FL, FM, TL and TM) and knee OA/non-OA binary classification tasks with sufficient data. Some additional OARSI features are not considered at all, such as medial tibial attrition, medial tibial sclerosis, lateral femoral sclerosis, etc. In the future, more OARSI features could be studied to provide additional clinical advice. Currently, knee joints detection and grades prediction are separate steps. Future work will focus on investigating an end-to-end deep learning system by combining these steps. Moreover, Magnetic Resonance Imaging (MRI) images will be used in the feature, which contains more information. In conclusion, this study demonstrates the automatic grading of individual knee OA features. Despite it has some shortcomings, we believe that the proposed approach has potential to become a useful tool in clinical OA trials and provide better quantitative information for doctors.

## Supplementary Information

Supplementary Information.
