# Supplementary material for: A siamese network with adaptive gated feature fusion for individual knee OA features grades prediction
Source: Sci Rep. 2021 Aug 19;11:16833. doi: 10.1038/s41598-021-96240-8 (PMC8376929; doi:10.1038/s41598-021-96240-8)
Supplement: Supplementary file 1 — Supplementary Information. [file 41598_2021_96240_MOESM1_ESM.pdf]

Title: A Siamese Network with Adaptive Gated Feature Fusion for Individual Knee OA Features Grades Prediction

Author: Kang Wang[1,*], Xin Niu[1], Yong Dou[1], Dongxing Xie[2], and Tuo Yang[3]

Affiliations: [1]National Laboratory for Parallel and Distributed Processing, School of Computer, National University of Defense Technology, Changsha 410073, China
[2]Department of Orthopaedics, Xiangya Hospital, Central South University, Changsha 410008, China
[3]Department of Health Management Center, Xiangya Hospital, Central South University, Changsha 410008, China
[*]Corresponding Author, E-mail: wangkang@nudt.edu.cn

Supplementary information

The training process of knee joints localization model

(1) **Labeling true bounding boxes of knee joint regions for generating training samples.** We randomly select 5448 single-knee images from the OAI dataset's baseline month to manually label six key points as shown in supplementary Figure 2. These 5448 single-knee images are labeled with real bounding boxes of knee joint regions as follows: As for each image, according to its key points manually labeled, we consider the average values of the horizontal and vertical coordinates of the six points as the horizontal and vertical coordinates of the center point. The difference between the maximum and minimum abscissas of the six key points is regarded as the knee joint width. The ordinate of the center point adds 0.65 times of the knee joint width and subtracts 0.65 times of the knee joint width, which is as the height of the ground truth of the bounding box. Meanwhile, the abscissa of the center point adds 0.65 times of the knee joint width and subtracts 0.65 times of the knee joint width, which is as the width of the ground truth of the bounding box. The red bounding box is the true bounding box of the knee joint as the supplementary Figure 2 shown. 5448 single-knee images are randomly divided into 4086 images to generate the training samples for the network of knee joints localization, and the remaining 1362 images to verify that method.

(2) **The training of the first-level network.** Firstly, we need to generate training samples of the first-level network. For each of 4086 images, we randomly select bounding boxes on the image and compare the Intersection Over Union (IOU) with its ground truth. For each selected area, if $IOU \geq 0.65$, it is a positive sample. If $0.4 \leq IOU < 0.65$, it is a partial sample. If $IOU < 0.3$, it is a negative sample. All generated training samples are resized into $48 \times 48 \times 3$ and augmented by mirror operations. In our experiments, the generated training samples for the first-level network contain 40131 positive samples, 220166 negative samples and 39363 partial samples. Then these training samples are fed into the first-level network, producing multiple sets of one-dimensional vector about knee/non-knee classification and 4-dimensional bounding box regression vectors of candidate knee joint regions. The training of the first-level network is completed until it reaches the setting epoch number. Specific training settings are described in implementation details of knee joint detection model. Finally, the trained model of the first-level network is obtained.

(3) **The training of the second-level network.** The training samples of the second-level network also need to be acquired firstly. Each of the 4086 single-knee images is scaled with different scales to produce the image pyramid. The image pyramid is fed into the trained first-level network, generating some candidate knee joint regions. These candidate areas are compared with their ground truth according to IOU. For each candidate, if $IOU \geq 0.65$, it is regarded as a positive sample. If $0.4 \leq IOU < 0.65$, it is considered as a partial sample. If $IOU < 0.3$, it is a negative sample. In addition, we randomly select bounding boxes from 4086 knee images and compare them with their ground truth by IOU. The selected areas with $IOU \geq 0.65$ are used as the training samples for six key points detection. All training samples are scaled to $48 \times 48 \times 3$ and the positive, negative and partial samples are augmented by mirror operations. In the end, generated training samples for the second-level network include 18450 positive samples, 7151 negative samples, 4993 partial samples and 20132 samples for key points localization. Some generated training samples of the knee joints localization model are shown in supplementary Figure 3. Then these training samples are transmitted into the second-level network, outputting multiple one-dimensional vectors about knee/non-knee classification, 4-dimensional bounding box regression vectors of candidate knee joint regions and 12-dimensional key points regression vectors. It trains until the setting epoch is met. Finally, the trained model of the second-level network is preserved.

Table 1. Description of the OAI dataset used in grading individual knee OA features.

Dataset	Grade	KL	FL	FM	TL	TM	JSN-L	JSN-M
OAI (Train: 13472)	0	1687	8046	6968	8247	4796	11627	6445
	1	1928	3171	3034	3442	6274	809	3901
	2	5770	1160	1406	816	1420	684	2356
	3	3160	1095	2064	967	982	352	770
	4	927	-	-	-	-	-	-
OAI (Val: 2732)	0	354	1612	1432	1676	993	2390	1314
	1	379	659	592	718	1230	155	753
	2	1168	257	309	152	316	127	501
	3	641	204	399	186	193	60	164
	4	190	-	-	-	-	-	-
OAI (Test: 8061)	0	1007	4830	4182	4847	2911	6949	3822
	1	1137	1859	1812	2121	3674	523	2305
	2	3460	669	813	461	870	387	1456
	3	1894	703	1254	632	606	202	478
	4	563	-	-	-	-	-	-

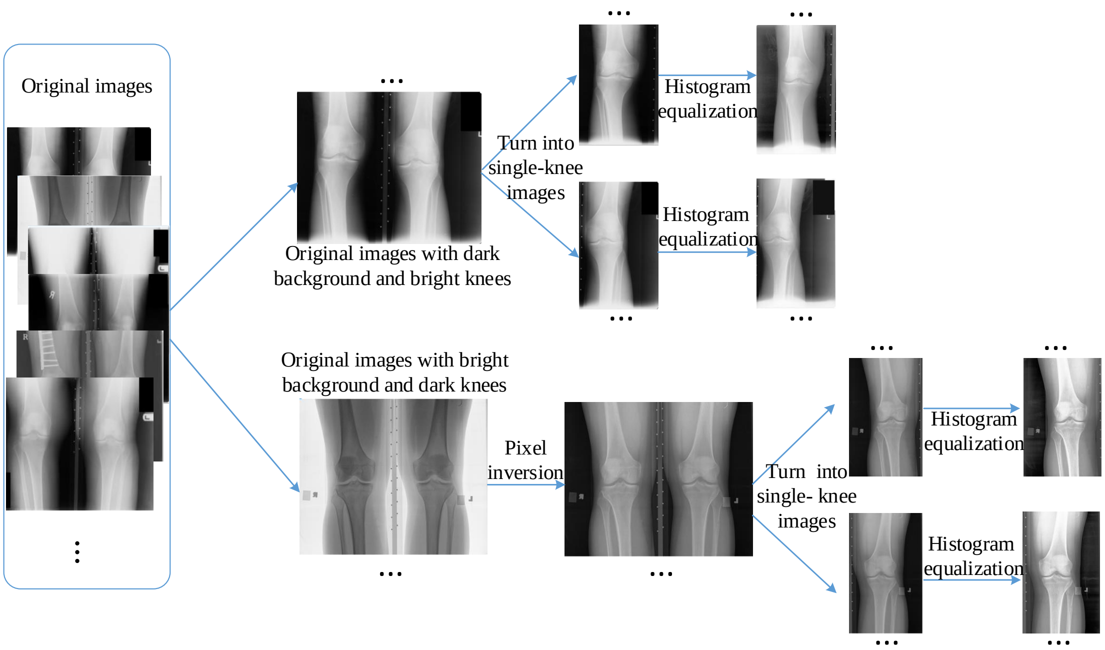

Figure 1. The specific process of data preprocessing.

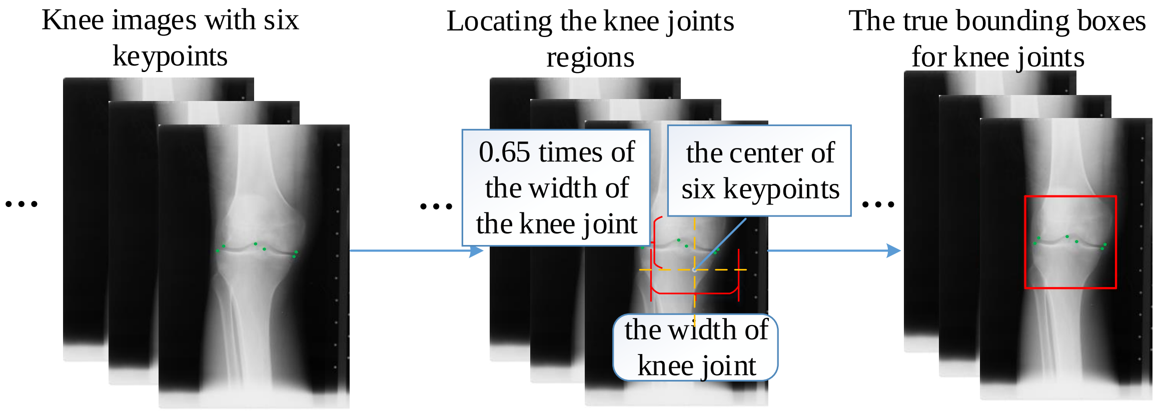

Figure 2. The process of generating true bounding boxes for the knee joints localization model.

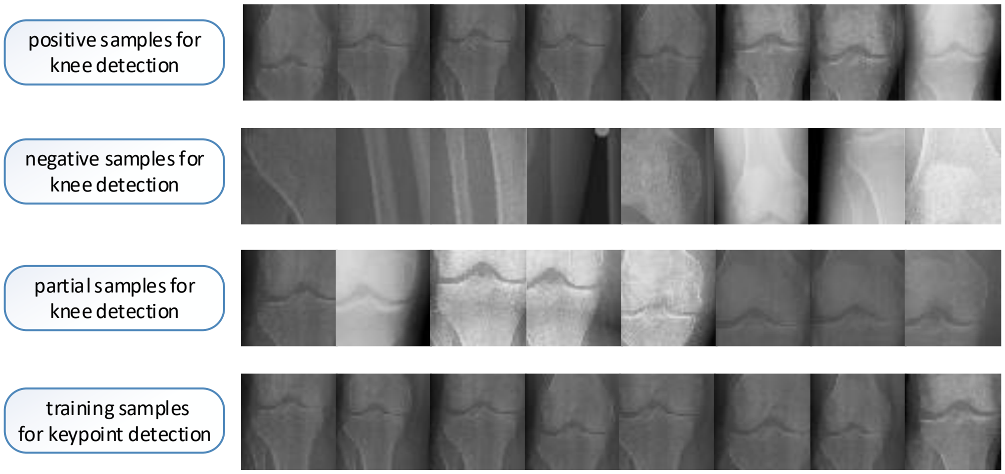

Figure 3. The examples of generated training samples for the knee joints localization model.

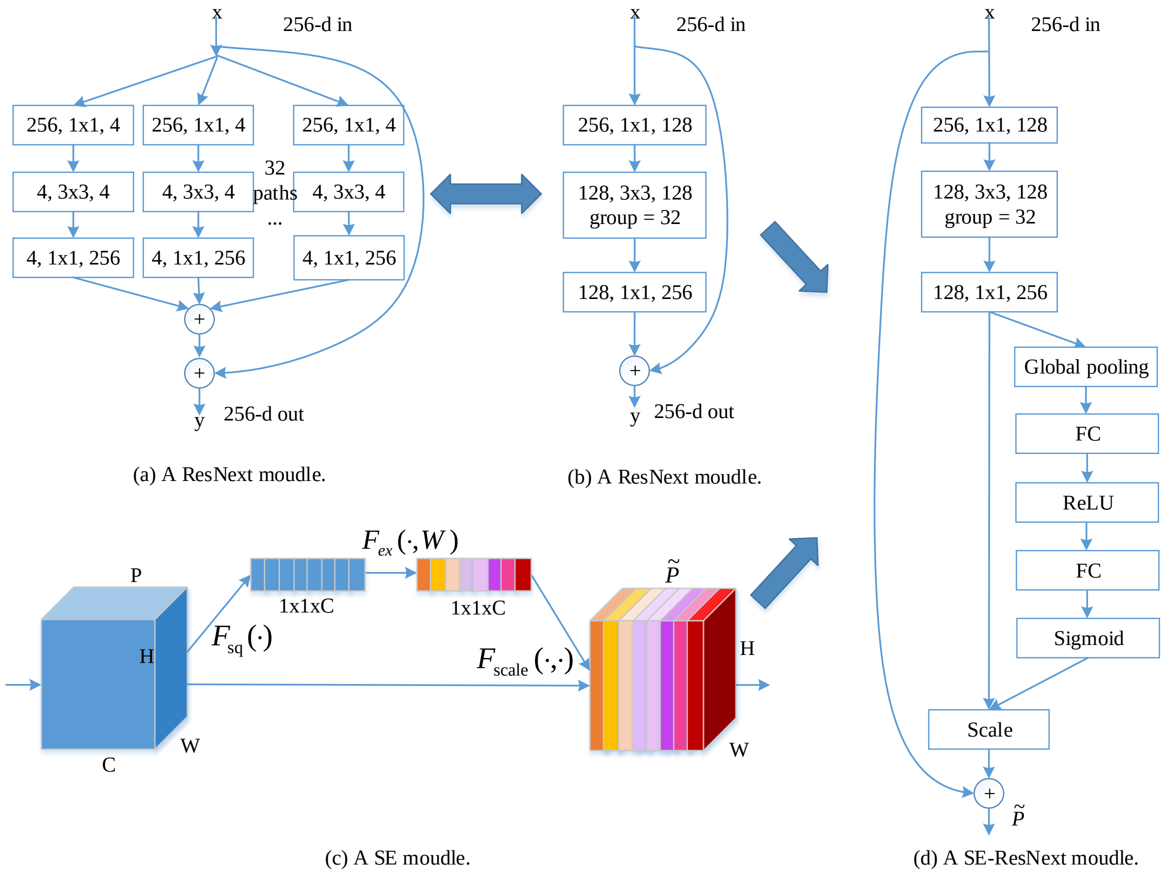

Figure 4. The basic SE-ResNext module.

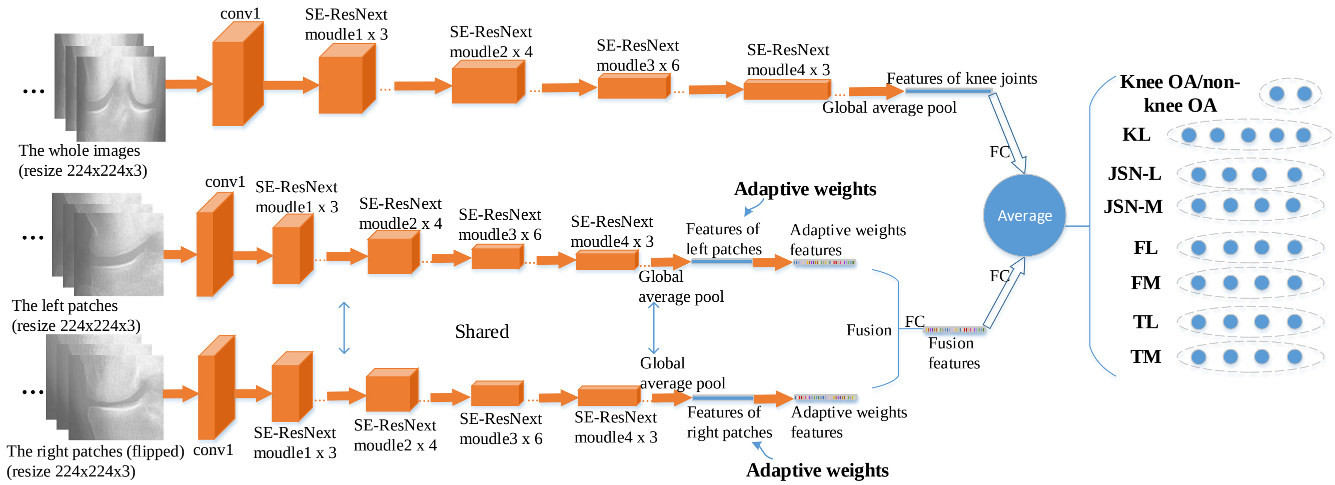

Figure 5. Our ensemble model of SE-ResNext50-32x4d and SE-ResNext50- 32x4d-based Siamese network for eight tasks. (The input of the SE-ResNext50-32x4d model is the whole knee joint area images, which are equally divided into two patches and input into the SE-ResNext50- 32x4d-based Siamese network.)

Table 2. Description of the MOST dataset used in grading individual knee OA features.

Dataset	Grade	KL	FL	FM	TL	TM	JSN-L	JSN-M
MOST (Train: 10244)	0	4180	7838	6848	7589	5489	9151	6188
	1	1516	1129	1079	1653	3125	429	1748
	2	1733	729	887	536	1093	401	1572
	3	1926	548	1430	466	537	263	736
	4	889	-	-	-	-	-	-
MOST (Val: 2048)	0	833	1530	1344	1506	1099	1832	1238
	1	306	244	218	320	630	82	348
	2	348	168	197	124	218	90	310
	3	385	106	289	98	101	44	152
	4	176	-	-	-	-	-	-
MOST (Test: 6162)	0	2519	4665	4113	4425	3390	5480	3840
	1	916	750	662	1098	1786	263	983
	2	1051	406	485	375	623	277	866
	3	1154	341	902	264	363	142	473
	4	522	-	-	-	-	-	-

Table 3. Performance comparison about Kappa and MSE between the proposed method and other methods on the OAI dataset.

Methods \ Kappa	knee OA/non-knee OA	KL	FL	FM	TL	TM	JSN-L	JSN-M
Antony et al., 2017	-	0.2428	-	-	-	-	-	-
Tiulpin et al., 2018	-	0.6291	-	-	-	-	-	-
Chen et al., 2019	-	0.8209	-	-	-	-	-	-
Mikhaylichenko et al., 2021	-	0.8216	-	-	-	-	-	-
SE-ResNet-50	-	0.8233	0.6961	0.7516	0.7401	0.7266	0.8023	0.8289
SE-ResNext50-32x4d	-	0.8439	0.7267	0.7769	0.7764	0.7540	0.8090	0.8340
Ensemble	-	0.8499	0.7263	0.7797	0.7762	0.7619	**0.8196**	**0.8463**
Ours	0.7140	0.8590	0.7424	0.7923	0.7796	0.7802	0.8080	0.8347
Ours (Ens.)	**0.7363**	**0.8666**	**0.7488**	**0.7952**	**0.7869**	**0.7829**	0.8071	0.8383
**Methods \ MSE**	**knee OA/non-knee OA**	**KL**	**FL**	**FM**	**TL**	**TM**	**JSN-L**	**JSN-M**
Antony et al., 2017	-	1.004	-	-	-	-	-	-
Tiulpin et al., 2018	-	0.7147	-	-	-	-	-	-
Chen et al., 2019	-	0.3655	-	-	-	-	-	-
Mikhaylichenko et al., 2021	-	0.4045	-	-	-	-	-	-
SE-ResNet-50	-	0.3955	0.5936	0.6364	0.4187	0.4129	0.1757	0.2950
SE-ResNext50-32x4d	-	0.3580	0.5071	0.5477	0.3910	0.3738	0.1671	0.2830
Ensemble	-	0.3408	0.5169	0.5493	0.3833	0.3598	**0.1580**	**0.2631**
Ours	0.1152	0.3418	**0.4689**	0.5074	0.3599	0.3588	0.1733	0.2878
Ours (Ens.)	**0.1040**	**0.3104**	0.4695	**0.5033**	**0.3397**	**0.3421**	0.1734	0.2812

Table 4. Performance comparison about Kappa and MSE between the proposed method and other methods on the MOST dataset.

Methods \ Kappa	knee OA/non-knee OA	KL	FL	FM	TL	TM	JSN-L	JSN-M
Antony et al., 2017	-	0.5720	-	-	-	-	-	-
Tiulpin et al., 2018	-	0.8460	-	-	-	-	-	-
Chen et al., 2019	-	0.9235	-	-	-	-	-	-
Mikhaylichenko et al., 2021	-	0.8979	-	-	-	-	-	-
SE-ResNet-50	-	0.9197	0.6571	0.7594	0.6313	0.7382	0.8130	0.8696
SE-ResNext50-32x4d	-	0.9219	0.7329	0.7902	0.7283	0.7721	0.8263	0.8768
Ensemble	-	0.9261	0.7128	0.7880	0.7043	0.7720	0.8293	**0.8792**
Ours	0.8493	0.9284	**0.7538**	**0.8071**	**0.7420**	0.7840	**0.8316**	0.8730
Ours (Ens.)	**0.8540**	**0.9289**	0.7222	0.7945	0.7252	**0.7935**	0.8187	0.8747
**Methods \ MSE**	**knee OA/non-knee OA**	**KL**	**FL**	**FM**	**TL**	**TM**	**JSN-L**	**JSN-M**
Antony et al., 2017	-	2.0141	-	-	-	-	-	-
Tiulpin et al., 2018	-	0.6480	-	-	-	-	-	-
Chen et al., 2019	-	0.3035	-	-	-	-	-	-
Mikhaylichenko et al., 2021	-	0.4062	-	-	-	-	-	-
SE-ResNet-50	-	0.3082	0.4942	0.5857	0.4622	0.3724	0.1522	0.2486
SE-ResNext50-32x4d	-	0.3019	0.4177	0.5377	0.3513	0.3466	0.1422	0.2356
Ensemble	-	0.2859	0.4338	0.5310	0.3768	0.3358	0.1394	**0.2309**
Ours	0.0742	**0.2746**	**0.3746**	**0.4734**	**0.3252**	0.3028	**0.1366**	0.2361
Ours (Ens.)	**0.0716**	0.2786	0.4120	0.5021	0.3403	**0.3020**	0.1452	0.2314

Table 5. Performance comparison between the proposed method and other methods on the MOST dataset. (This is trained on the OAI dataset and tested on the MOST dataset.)

Methods \ top1	knee OA/non-knee OA	KL	FL	FM	TL	TM	JSN-L	JSN-M
Antony et al., 2017	-	18.44%	-	-	-	-	-	-
Tiulpin et al., 2018	-	61.98%	-	-	-	-	-	-
Chen et al., 2019	-	63.95%	-	-	-	-	-	-
Mikhaylichenko et al., 2021	-	66.87%	-	-	-	-	-	-
SE-ResNet-50	-	64.39%	78.18%	76.96%	74.70%	72.46%	92.27%	79.10%
SE-ResNext50-32x4d	-	66.42%	**80.63**%	77.15%	76.79%	71.52%	**93.04**%	77.12%
Ensemble	-	66.01%	80.28%	77.78%	76.64%	72.90%	92.98%	78.86%
Ours	**87.51**%	**67.47**%	79.71%	79.26%	79.46%	74.34%	92.38%	77.78%
Ours (Ens.)	87.04%	67.35%	80.28%	**80.03**%	**79.78**%	**75.35**%	92.75%	**79.37**%
Methods \ top±1	-	KL	FL	FM	TL	TM	JSN-L	JSN-M
Antony et al., 2017	-	52.64%	-	-	-	-	-	-
Tiulpin et al., 2018	-	87.80%	-	-	-	-	-	-
Chen et al., 2019	-	**95.13**%	-	-	-	-	-	-
Mikhaylichenko et al., 2021	-	89.21%	-	-	-	-	-	-
SE-ResNet-50	-	88.71%	**94.88**%	93.52%	96.21%	97.12%	98.00%	97.84%
SE-ResNext50-32x4d	-	90.98%	94.68%	93.50%	96.16%	96.96%	98.17%	**98.13**%
Ensemble	-	89.87%	94.81%	93.72%	96.35%	97.19%	**98.22**%	**98.13**%
Ours	-	94.71%	94.58%	93.76%	96.14%	97.74%	98.11%	97.53%
Ours (Ens.)	-	94.14%	94.87%	**94.09**%	**96.45**%	**97.82**%	98.09%	97.91%

Table 6. Performance comparison about Kappa and MSE between the proposed method and other methods on the MOST dataset. (This is trained on the OAI dataset and tested on the MOST dataset.)

Methods \ Kappa	knee OA/non-knee OA	KL	FL	FM	TL	TM	JSN-L	JSN-M
Antony et al., 2017	-	0.0749	-	-	-	-	-	-
Tiulpin et al., 2018	-	0.8017	-	-	-	-	-	-
Chen et al., 2019	-	0.8621	-	-	-	-	-	-
Mikhaylichenko et al., 2021	-	0.8277	-	-	-	-	-	-
SE-ResNet-50	-	0.8196	0.7289	0.7740	0.7225	0.7429	0.8016	0.8420
SE-ResNext50-32x4d	-	0.8419	0.7290	0.7782	0.7354	0.7564	0.8152	0.8353
Ensemble	-	0.8332	0.7350	0.7850	0.7407	0.7605	**0.8199**	0.8458
Ours	**0.7527**	**0.8753**	0.7319	0.7883	0.7397	0.7770	0.8103	0.8352
Ours (Ens.)	0.7442	0.8700	**0.7411**	**0.8007**	**0.7512**	**0.7874**	0.8119	**0.8485**
Methods \ MSE	knee OA/non-knee OA	KL	FL	FM	TL	TM	JSN-L	JSN-M
Antony et al., 2017	-	2.2534	-	-	-	-	-	-
Tiulpin et al., 2018	-	0.7681	-	-	-	-	-	-
Chen et al., 2019	-	0.5073	-	-	-	-	-	-
Mikhaylichenko et al., 2021	-	0.6693	-	-	-	-	-	-
SE-ResNet-50	-	0.6963	0.4559	0.5368	0.3963	0.3802	0.1743	0.3070
SE-ResNext50-32x4d	-	0.6101	0.4410	0.5455	0.3844	0.4058	0.1569	0.3161
Ensemble	-	0.6453	0.4410	0.5221	0.3743	0.3818	**0.1547**	**0.2983**
Ours	**0.1249**	**0.4892**	0.4540	0.5208	0.3611	0.3513	0.1648	0.3274
Ours (Ens.)	0.1296	0.5062	**0.4339**	**0.4912**	**0.3453**	**0.3375**	0.1628	0.2990

Table 7. Performance Comparison of ablation experiments on the OAI dataset.

Methods \ top1	knee OA/non-knee OA	KL	FL	FM	TL	TM	JSN-L	JSN-M
baseline (SE-ResNext50-32x4d)	-	75.74%	72.75%	72.91%	76.79%	71.69%	**91.48**%	79.92%
baseline+Siamese	-	73.66%	74.42%	74.71%	**77.48**%	72.11%	89.83%	76.79%
baseline+Siamese+knee OA/non-knee OA task	**89.11**%	75.97%	74.69%	74.51%	76.80%	**73.08**%	91.12%	78.71%
Ours (baseline+Siamese+knee OA/non-knee OA task + adaptively gated feature fusion)	88.48%	**76.32**%	**74.80**%	**74.73**%	76.81%	72.73%	91.11%	**80.00**%
Methods \ top±1	knee OA/non-knee OA	KL	FL	FM	TL	TM	JSN-L	JSN-M
baseline (SE-ResNext50-32x4d)	-	96.22%	94.52%	94.02%	96.07%	97.43%	**98.02**%	**97.90**%
baseline+Siamese	-	96.79%	95.11%	94.02%	**97.39**%	97.74%	97.52%	97.32%
baseline+Siamese+knee OA/non-knee OA task	-	**97.12**%	**95.66**%	**94.86**%	97.15%	**97.99**%	97.93%	97.75%
Ours (baseline+Siamese+knee OA/non-knee OA task + adaptively gated feature fusion)	-	96.63%	95.31%	94.59%	96.91%	97.67%	97.89%	97.75%

Table 8. Performance Comparison of ablation experiments on the MOST dataset.

top1 / Methods	knee OA/non-knee OA	KL	FL	FM	TL	TM	JSN-L	JSN-M
baseline (SE-ResNext50-32x4d)	-	75.22%	78.98%	78.72%	78.04%	73.11%	93.43%	**84.16%**
baseline+Siamese	-	**77.04%**	79.93%	79.03%	79.07%	75.49%	93.07%	83.84%
baseline+Siamese+knee OA/non-knee OA task	92.53%	76.76%	80.69%	79.96%	**80.12%**	76.19%	92.83%	83.22%
Ours (baseline+Siamese+knee OA/non-knee OA task + adaptively gated feature fusion)	**92.58%**	76.94%	**82.05%**	**80.54%**	**80.12%**	**76.32%**	**93.54%**	83.77%
top±1 / Methods	-	KL	FL	FM	TL	TM	JSN-L	JSN-M
baseline (SE-ResNext50-32x4d)	-	98.20%	95.16%	93.28%	96.45%	97.84%	98.26%	98.26%
baseline+Siamese	-	**98.64%**	95.07%	92.71%	96.54%	97.52%	97.99%	**98.33%**
baseline+Siamese+knee OA/non-knee OA task	-	97.89%	95.47%	**94.22%**	96.51%	97.63%	98.25%	97.97%
Ours (baseline+Siamese+knee OA/non-knee OA task + adaptively gated feature fusion)	-	98.59%	**95.55%**	94.06%	**96.87%**	**98.15%**	**98.33%**	98.21%

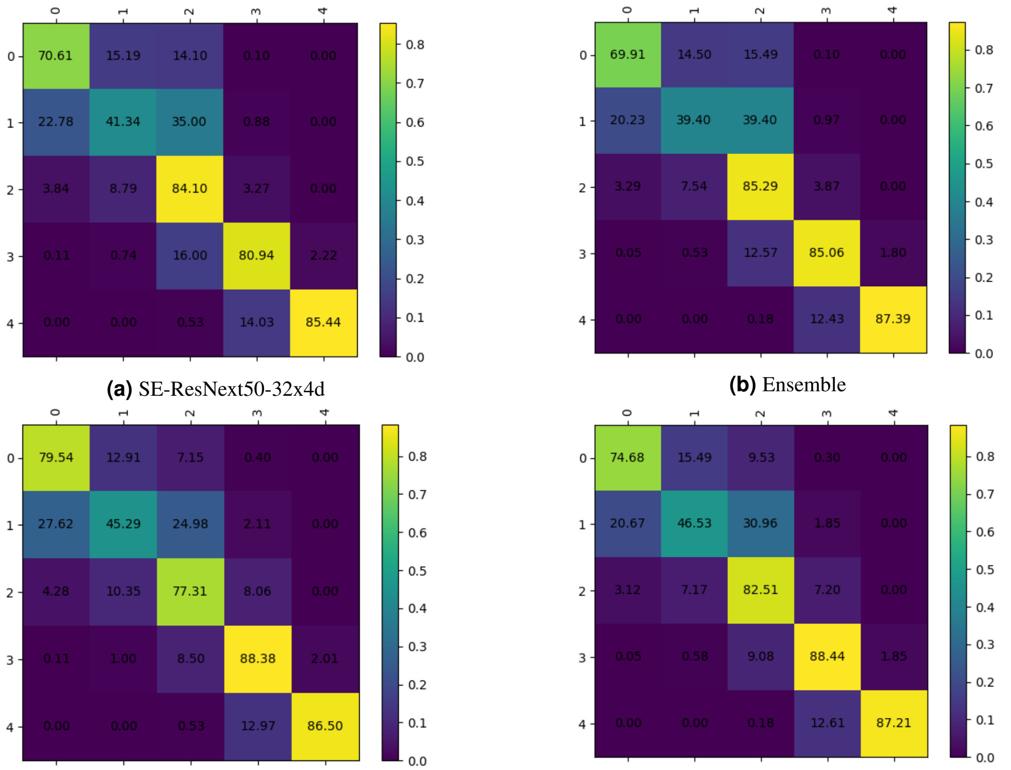

(a) SE-ResNext50-32x4d

(b) Ensemble

(c) Ours

(d) Ours (Ens.)

Figure 6. Confusion matrices for KL grades prediction tasks under different methods on the OAI dataset.

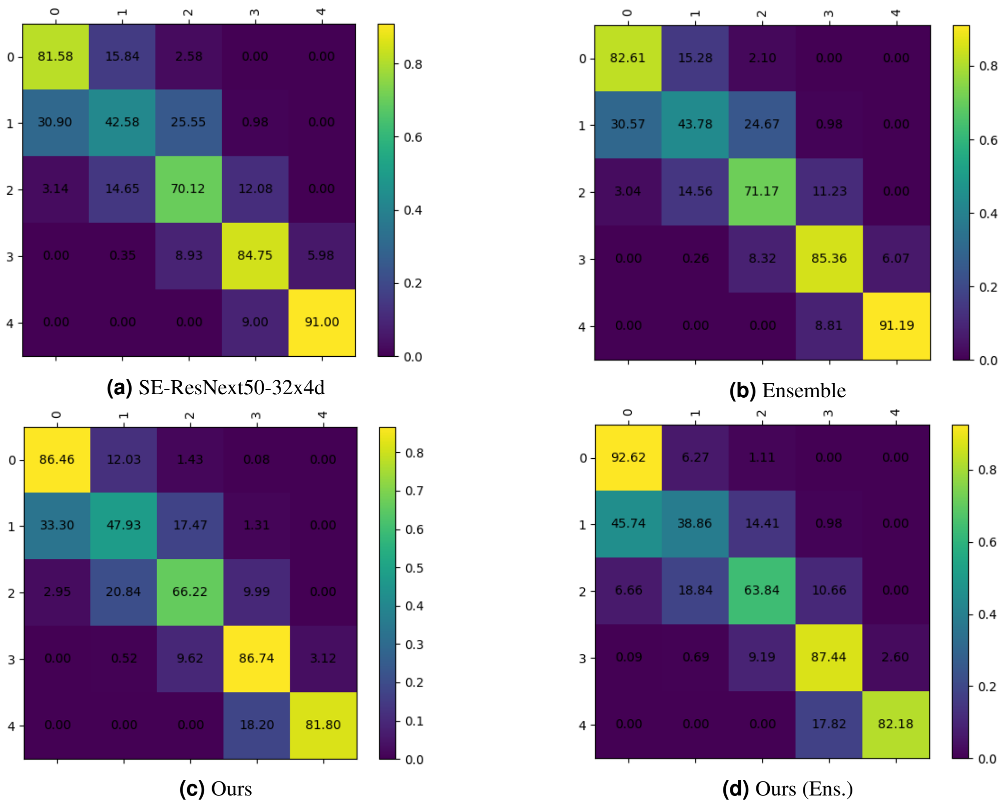

(a) SE-ResNext50-32x4d

(b) Ensemble

(c) Ours

(d) Ours (Ens.)

Figure 7. Confusion matrices for KL grades prediction tasks under different methods on the MOST dataset.

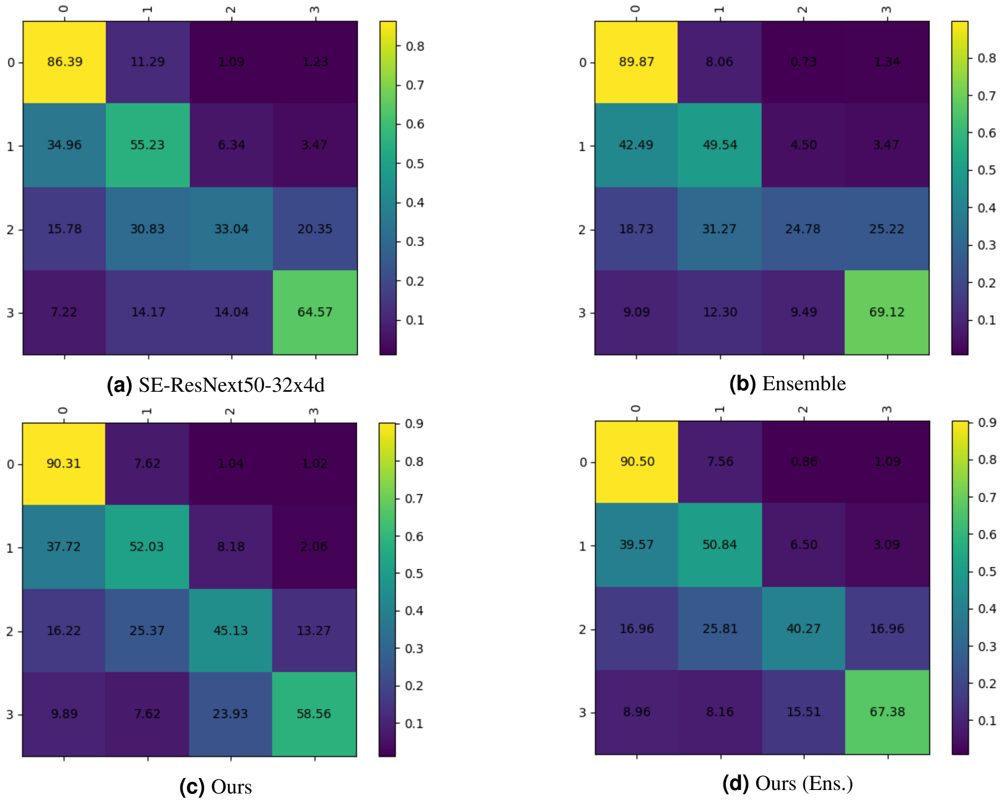

(a) SE-ResNext50-32x4d

(b) Ensemble

(c) Ours

(d) Ours (Ens.)

Figure 8. Confusion matrices for FL grades prediction tasks under different methods on the OAI dataset.

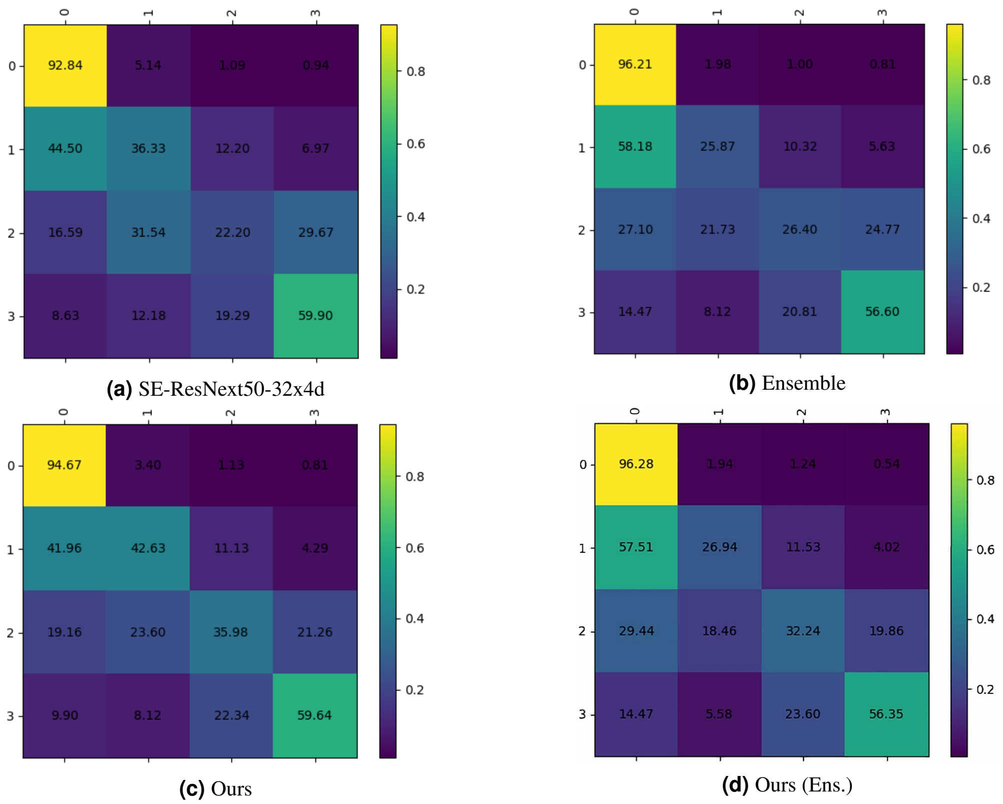

(a) SE-ResNext50-32x4d **(b)** Ensemble

(c) Ours **(d)** Ours (Ens.)

Figure 9. Confusion matrices for FL grades prediction tasks under different methods on the MOST dataset.

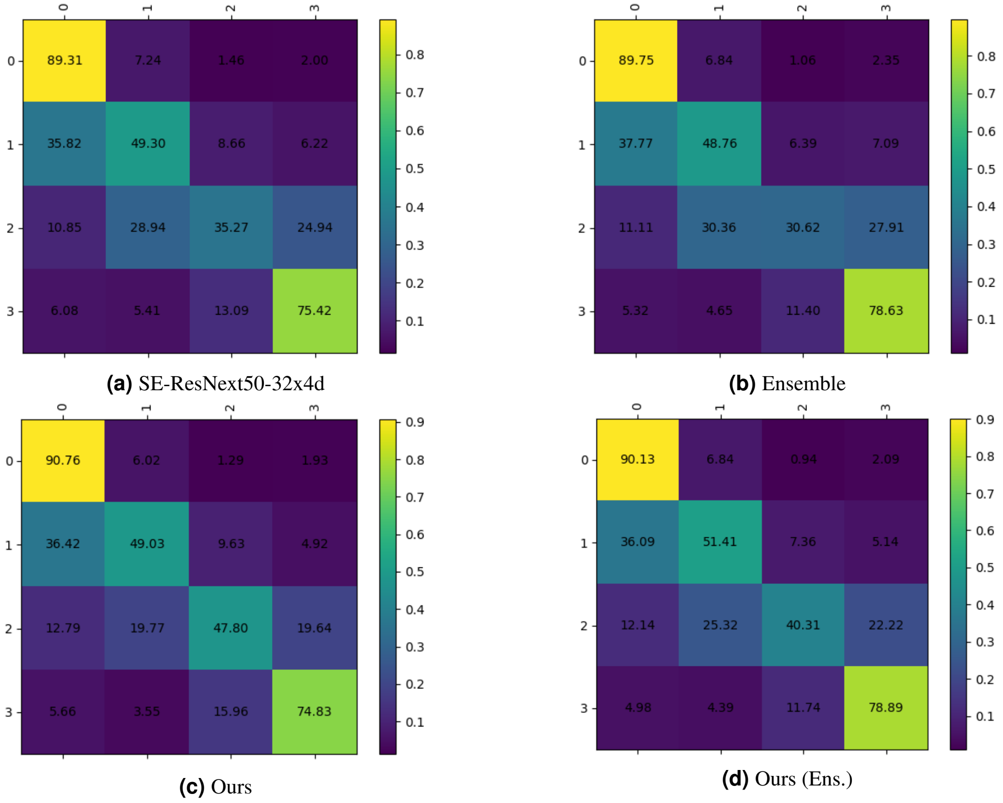

(a) SE-ResNext50-32x4d **(b)** Ensemble

(c) Ours **(d)** Ours (Ens.)

Figure 10. Confusion matrices for FM grades prediction tasks under different methods on the OAI dataset.

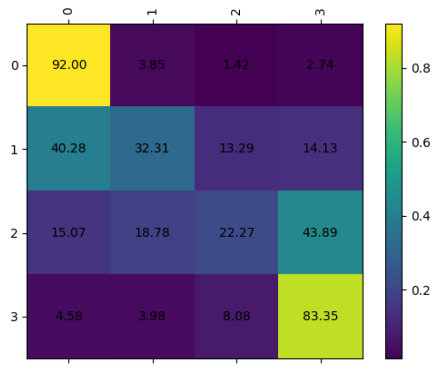

(a) SE-ResNext50-32x4d

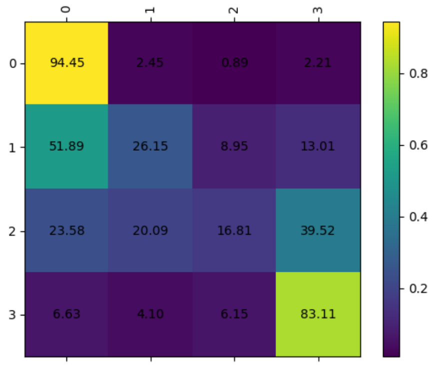

(b) Ensemble

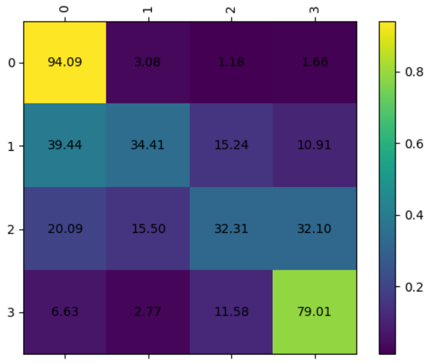

(c) Ours

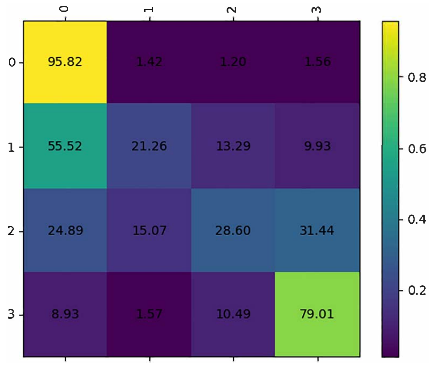

(d) Ours (Ens.)

Figure 11. Confusion matrices for FM grades prediction tasks under different methods on the MOST dataset.

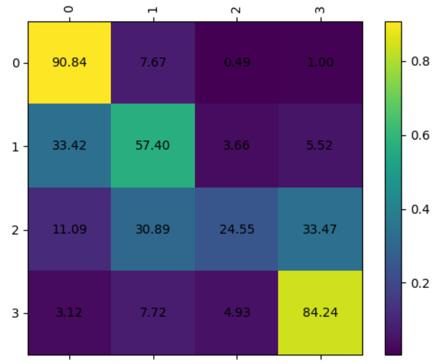

(a) SE-ResNext50-32x4d

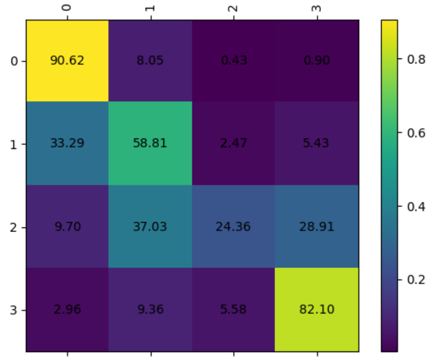

(b) Ensemble

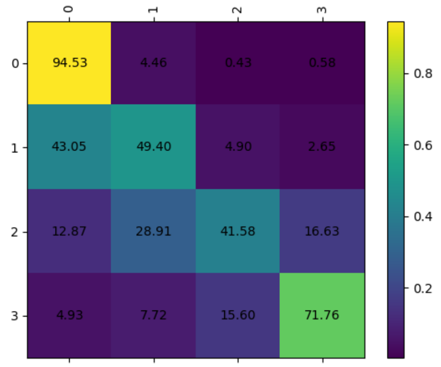

(c) Ours

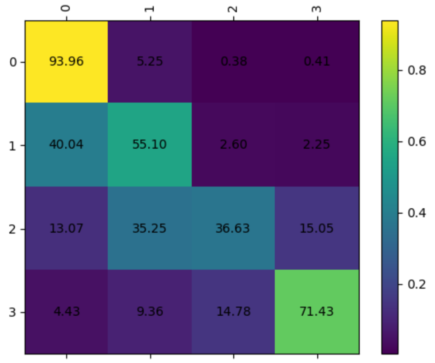

(d) Ours (Ens.)

Figure 12. Confusion matrices for TL grades prediction tasks under different methods on the OAI dataset.

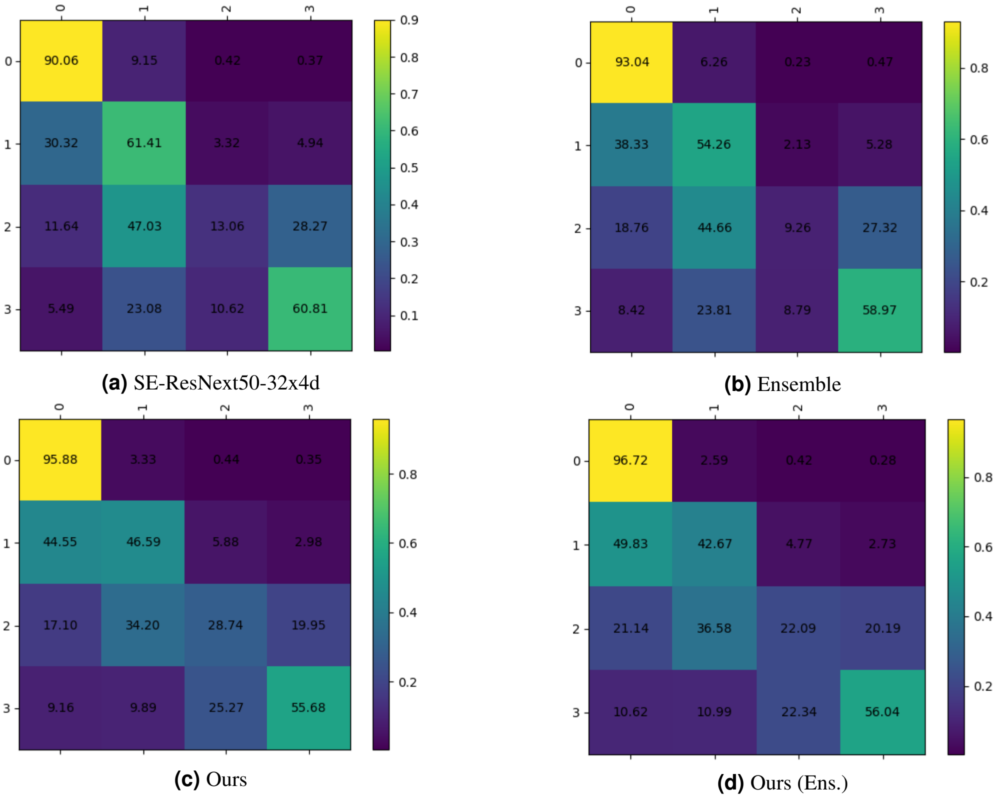

(a) SE-ResNext50-32x4d **(b)** Ensemble

(c) Ours **(d)** Ours (Ens.)

Figure 13. Confusion matrices for TL grades prediction tasks under different methods on the MOST dataset.

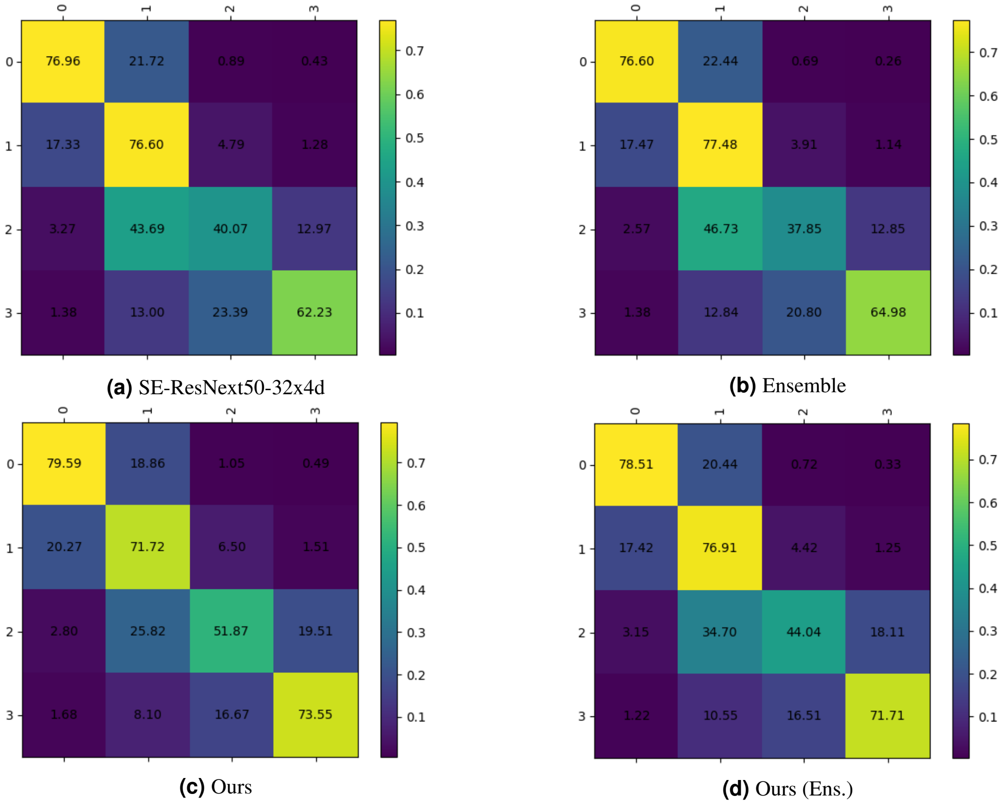

(a) SE-ResNext50-32x4d **(b)** Ensemble

(c) Ours **(d)** Ours (Ens.)

Figure 14. Confusion matrices for TM grades prediction tasks under different methods on the OAI dataset.

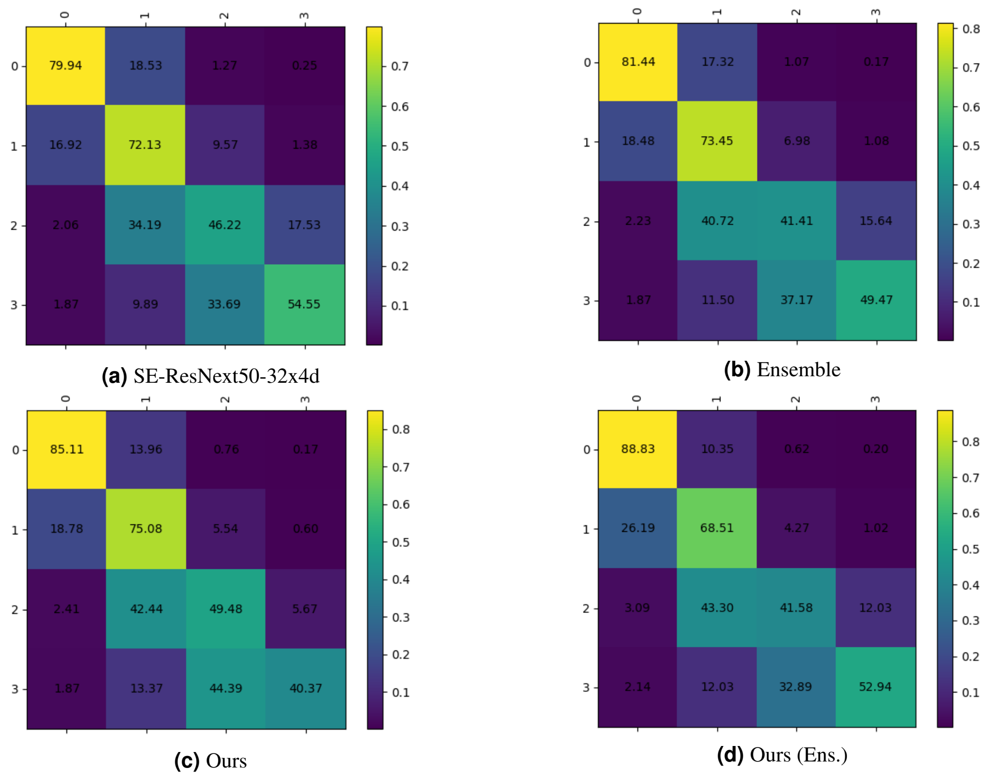

(a) SE-ResNext50-32x4d **(b)** Ensemble **(c)** Ours **(d)** Ours (Ens.)

Figure 15. Confusion matrices for TM grades prediction tasks under different methods on the MOST dataset.

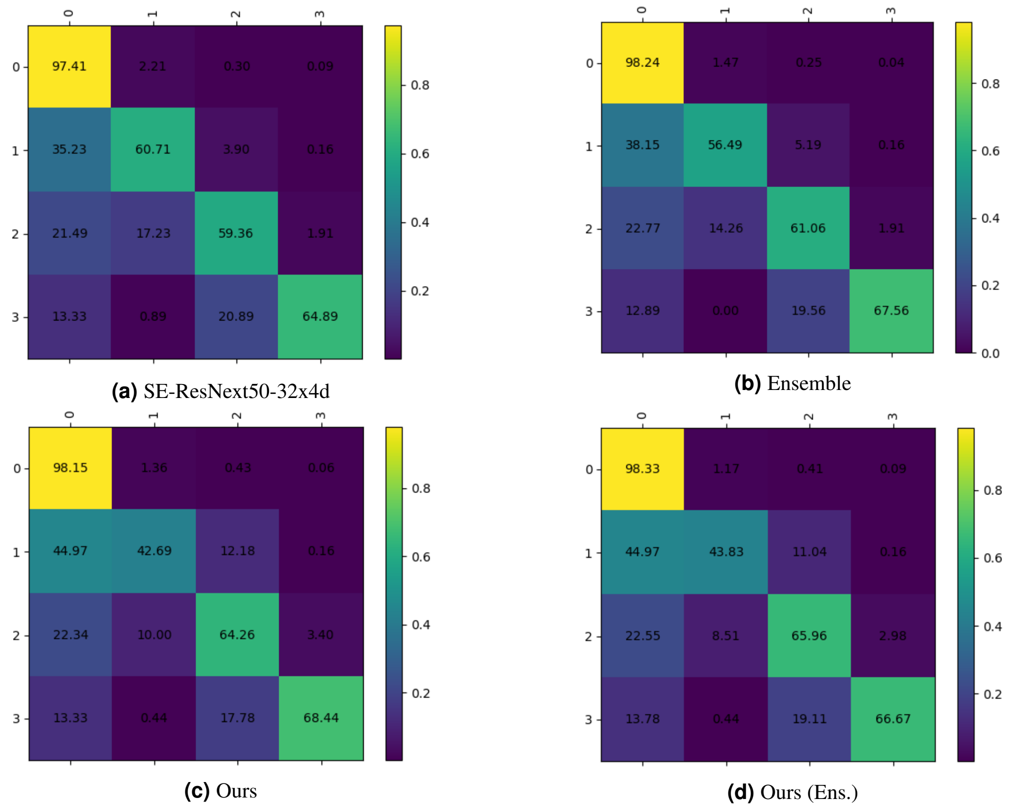

(a) SE-ResNext50-32x4d **(b)** Ensemble **(c)** Ours **(d)** Ours (Ens.)

Figure 16. Confusion matrices for JSN-L grades prediction tasks under different methods on the OAI dataset.

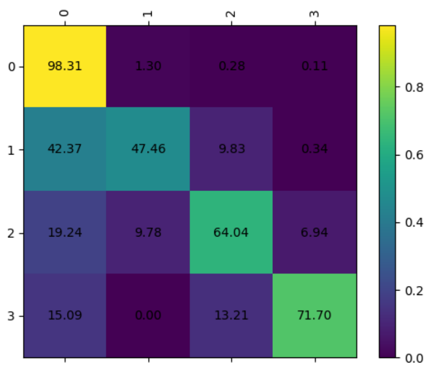

(a) SE-ResNext50-32x4d

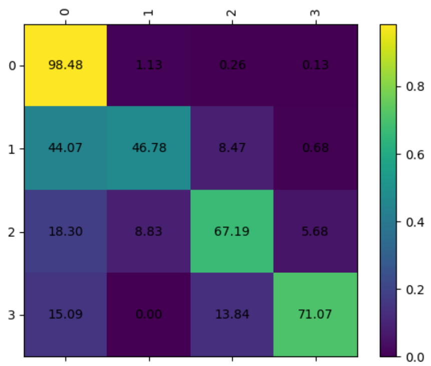

(b) Ensemble

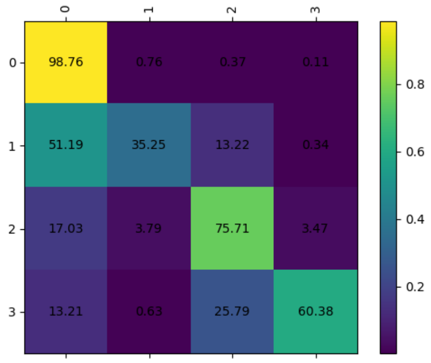

(c) Ours

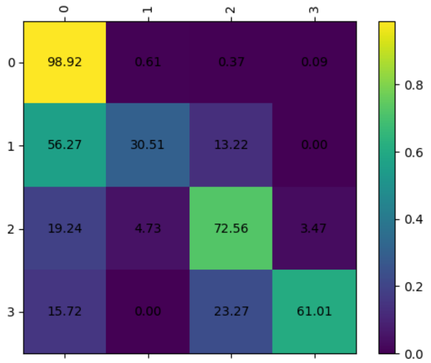

(d) Ours (Ens.)

Figure 17. Confusion matrices for JSN-L grades prediction tasks under different methods on the MOST dataset.

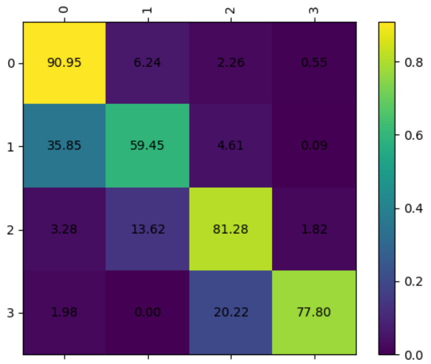

(a) SE-ResNext50-32x4d

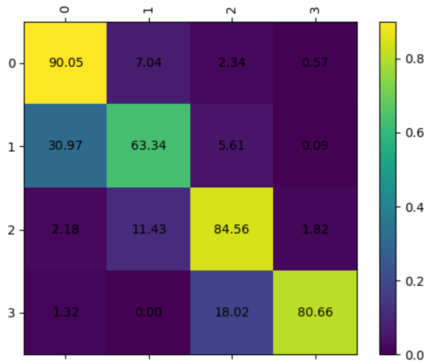

(b) Ensemble

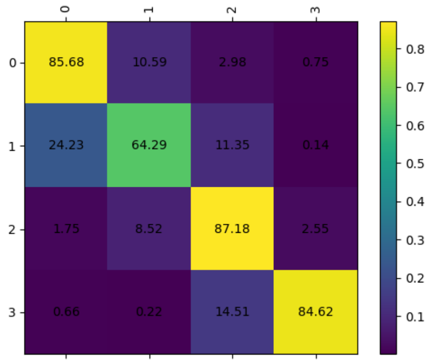

(c) Ours

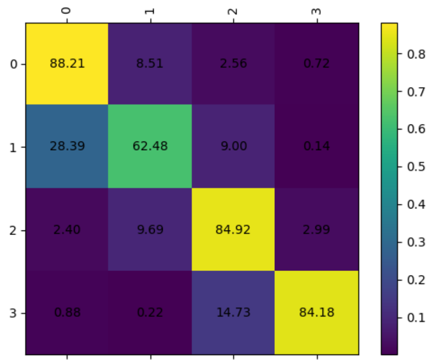

(d) Ours (Ens.)

Figure 18. Confusion matrices for JSN-M grades prediction tasks under different methods on the OAI dataset.

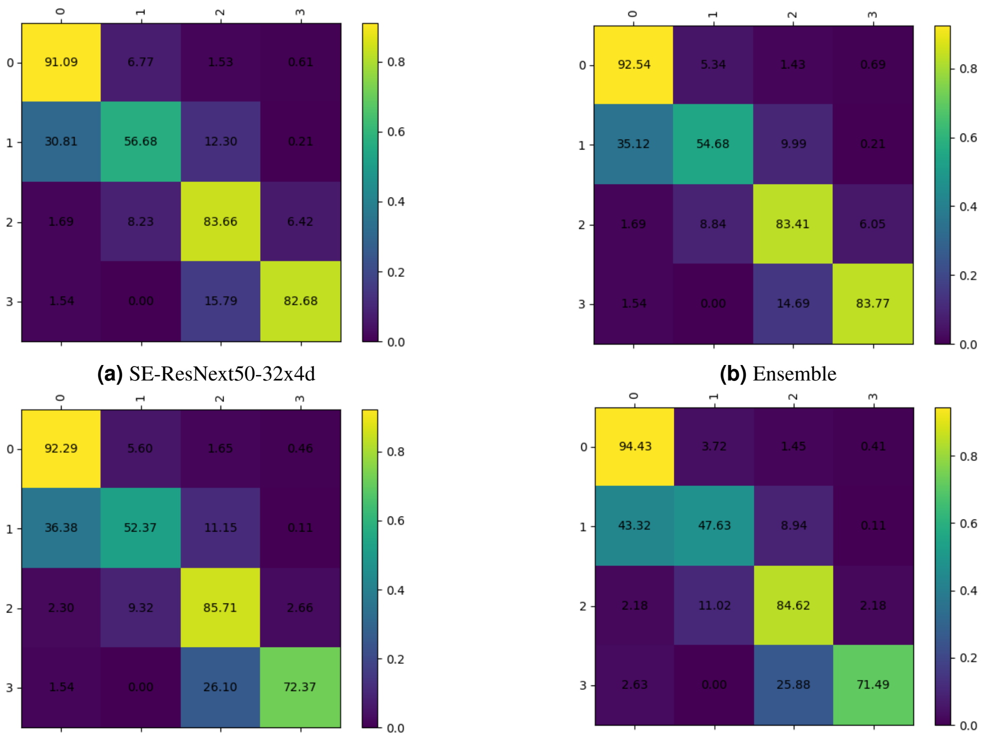

(a) SE-ResNext50-32x4d

(b) Ensemble

(c) Ours

(d) Ours (Ens.)

Figure 19. Confusion matrices for JSN-M grades prediction tasks under different methods on the MOST dataset.

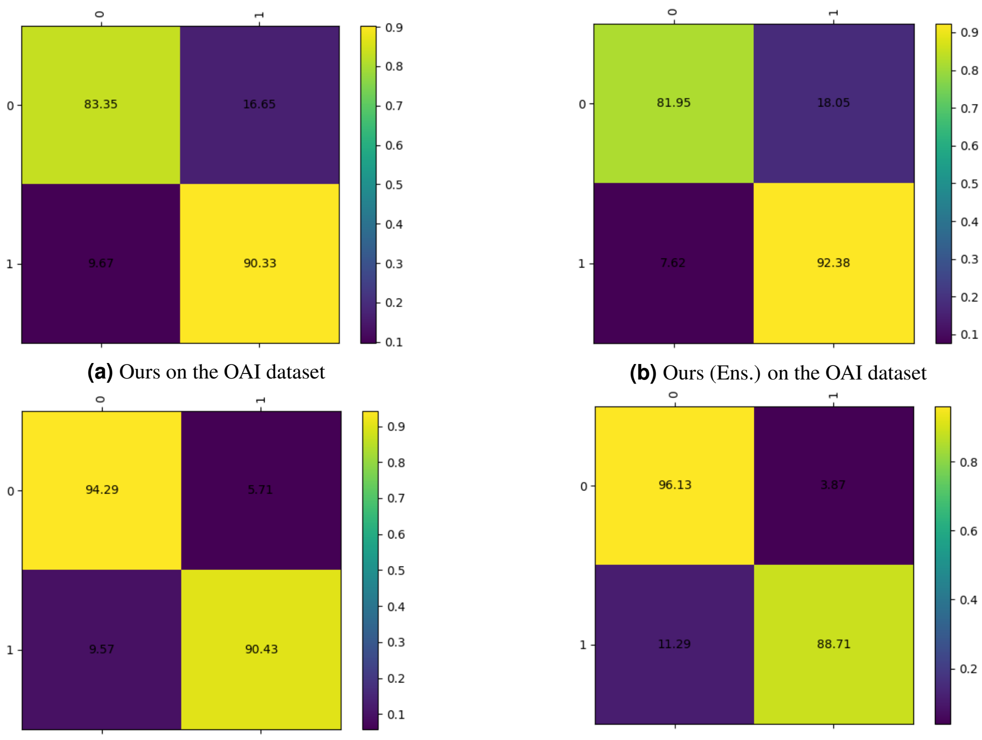

(a) Ours on the OAI dataset

(b) Ours (Ens.) on the OAI dataset

(c) Ours on the MOST dataset

(d) Ours (Ens.) on the MOST dataset

Figure 20. Confusion matrices for knee OA/non-knee OA classification tasks under different methods on the OAI and MOST datasets.
